# Self-Assembly of Au/TiO_2_ Sphere–Rod Mixtures into Binary Supraparticles

**DOI:** 10.1021/acsnano.6c12966

**Published:** 2026-09-25

**Authors:** Marianne Bijl, André F. V. Matias, Lukáš Kolácný, Erik Betz-Güttner, Martin Veselý, Marjolein Dijkstra, Jessi E. S. van der Hoeven, Alfons van Blaaderen

**Affiliations:** † Materials Chemistry and Catalysis, Debye Institute for Nanomaterials Science, 8125 Utrecht University, Universiteitsweg 99, 3584 CG Utrecht, The Netherlands; ‡ Soft Condensed Matter & Biophysics Group, Debye Institute for Nanomaterials Science, Utrecht University, Princetonplein 1, 3584 CC Utrecht, The Netherlands; § Department of Organic Technology, 52735 University of Chemistry and Technology Prague, Technická 5, 166 28 Prague, Czech Republic

**Keywords:** self-assembly, supraparticles, binary smectic, colloidal membranes, gold nanospheres, titania nanorods

## Abstract

Self-assembly of rod–sphere nanoparticle mixtures enables the design of advanced materials with anisotropic properties. While the phase behavior of rod–sphere mixtures has been explored in 2D and 3D bulk systems, it has remained unexplored in smaller 3D assemblies. Especially, when combining plasmonic and semiconductor nanoparticles, assemblies with defined particle arrangements offer unique optical and (photo)­catalytic properties. In this work, we achieved the assembly of binary 3D assemblies tunable in size and phase behavior. Specifically, we assembled semiconductor TiO_2_ nanorods and plasmonic Au nanospheres under spherical confinement and explored their phase behavior in experiments and simulations. Through evaporation-driven assembly of emulsion droplets, binary supraparticles were prepared with a phase tunable from colloidal membranes, consisting of single rod layers, to binary smectic phases, composed of alternating rod–sphere smectic layers. Colloidal membranes were obtained for a sphere-to-rod number ratio (*N*_Au_/*N*_TiO2_) of 5, while lowering this ratio to 0.6 resulted in a binary smectic ordering, as confirmed with advanced electron microscopy. Through numerical simulations, we show that the binary smectic phase is stable for 0 < *N*_Au_/*N*_TiO2_ ≤ 1 in agreement with experiments. Our binary supraparticle platform provides structures with multi-length scale control for applications in photocatalysis, energy storage, and sensing.

## Graphical abstract

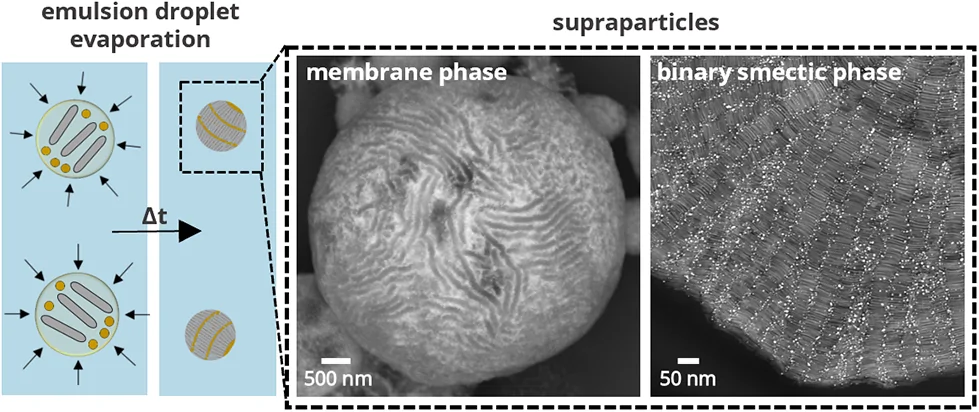

## Introduction

The assembly of nanoparticles into larger systems has shown great potential for the fabrication of advanced materials.^1^ In recent decades, scientists have used this bottom-up approach in which colloidal nanoparticles act as building blocks to design materials with multi-length scale property control, finding applications ranging from drug delivery^2–4^ to catalysis,^5–8^ sensors,^9–12^ and beyond.^13–15^ By combining two distinct types of nanoparticles, binary assemblies can be created with a rich class of structures and compositions. For example, by varying the nanoparticle size ratio, concentrations, and interparticle interactions, over 15 different binary superlattices can be constructed with well-defined stoichiometries.^16^ This structural tunability enables novel and improved collective properties that are unattainable in single-component assemblies. Distinct electronic properties were, for example, found in PbTe/Ag_2_Te binary assemblies, which exhibited up to ∼100-fold increased p-type conductivity compared to the sum of the individual components.^17^ The introduction of asymmetric shaped particles, such as anisotropic rods in binary rod–sphere mixtures, are interesting for introducing additional shape-dependent interactions.^18,19^ For instance, rod-shaped particles tend to align along a common director axis, promoting ordered structures similar to liquid crystals.^20,21^ Such structural alignment can further affect functional properties, such as optical birefringence and directional charge transport.

Binary rod–sphere mixtures can be assembled into various phases, among which the binary smectic phase and colloidal membrane phase exhibit the highest degree of order. In 1996, a theoretical study on hard rods and spheres predicted the formation of a binary smectic phase, consisting of alternating layers of rods and spheres.^22^ Experimental realization of the binary smectic phase was demonstrated by Adams et al. for rod-shaped fd viruses and polymer spheres, showing entropy-driven phase behavior.^23^ The binary smectic phase (also referred to as lamellar phase^23–25^ ) specifically consists of rod–sphere assemblies in which rods align in smectic layers alternating with a thin layer of spheres. Ye et al. coassembled colloidal rods and spheres into binary smectic superlattices for various rod–sphere combinations and with much lower aspect ratios than the fd viruses.^24^ To explain the observed phase behavior, attractions between the nanoparticles must have been present. Subsequently, Bakker et al. showed that a binary smectic phase can also be stable over a broad region of compositions and pressures for purely hard interparticle interactions and mapped out the phase diagram both in experiments and simulations.^26^ For systems with only rods, simulations^27,28^ and experiments^29,30^ have demonstrated that the smectic phase nucleates via a 2D rod layer onto which other layers nucleate and grow. However, the nucleation and growth mechanism of a binary smectic phase has not yet been reported, nor in experiment or theory. If 2D assemblies of rods also develop repulsions induced by fluctuations between the rod sheets, a phase of colloidal membranes is formed that does not progress into a binary smectic phase.^31^ Colloidal membranes consist of 2D sheets of one rod-length thick aligned rods. The 2D membranes were experimentally found by the Dogic group using fd viruses mixed with a nonadsorbing polymer acting as a depletant.^31,32^ As mentioned, this phase behavior is stabilized by repulsive interactions between the fluctuating membranes, promoting isolation.^31^ These repulsive interactions together with attractive depletion interactions are sensitive to the concentration of the depletion-inducing agent. If the concentration decreases under a certain threshold, the 2D membranes become unstable and form a 3D liquid crystalline phase.^33^

The assembly of rods and spheres in finite-sized 3D assemblies with intermediate length scales—in between 2D superlattices and 3D bulk systems—has remained unexplored as far as we know. This is unfortunate, as finite-sized 3D nanoparticle assemblies can exhibit unique magnetic,^12,34^ optical,^35–37^ and catalytic^5^ properties that are typically not observed at both nano- and bulk scale. Furthermore, spherical nanoparticle assemblies, known as supraparticles, can for instance exhibit Mie whispering gallery modes, allowing light to resonate along the surface.^35,37^ This enhances light–matter interactions that can lead to strongly nonlinear enhancement, such as lasing.^35^ Another intriguing finding is that hard spherical particles self-assembled under spherical confinement, even up to 100.000 particles or more, do not form the bulk equilibrium close-packed face-centered cubic crystal phase, but instead icosahedral assemblies.^38^ Thus, the phase behavior in smaller 3D assemblies can substantially deviate from that in bulk.

In this work, we report the formation of 3D binary supraparticles exhibiting tunable phase behavior from a colloidal membrane to a binary smectic phase for the first time. Specifically, plasmonic gold (Au) nanospheres and semiconductor titanium dioxide (TiO_2_) nanorods were used for their complementary optical and catalytic properties.^39^ First, we investigated the 2D and several thin layers of 3D self-assembly of an apolar sphere–rod Au/TiO_2_ dispersion on top of an aqueous surface in order to gain insights into the phase behavior without spherical confinement effects. Subsequently, we investigated the 3D self-assembly behavior under spherical confinement, where supraparticles with membrane and binary smectic phases were successfully formed by varying the particle number ratio (*N*_Au_/*N*_TiO2_). Furthermore, supraparticle formation via evaporation-driven self-assembly in emulsion droplets was followed in situ using polarized optical microscopy (POM),^40^ revealing a steep increased birefringence in later stages of the assembly process. The experiments are supported with molecular dynamics (MD) simulations to determine the phase diagram of our rod–sphere mixtures in bulk, confirming tunable phase behavior with *N*_Au_/*N*_TiO2_.

## Results

### The Effect of the Rod-to-Sphere Ratio on Binary 2D Assemblies

The Au nanospheres and TiO_2_ nanorods were synthesized via colloidal techniques obtained from literature (Figure 1a), as detailed in Experimental Methods. In short, oleylamine and oleic acid-capped single crystalline brookite TiO_2_ nanorods with a bare length of 49 ± 7 nm (mean ± polydispersity) and a bare diameter of 3.5 ± 0.6 nm were synthesized through a seed-mediated procedure.^41,42^ After an additional treatment to increase the ligand density,^43^ the interparticle surface-to-surface spacing was 2.7 ± 0.5 nm, corresponding to an effective aspect ratio of 8.3, which includes the ligands on the nanoparticles. Note that these spacings were measured on dried particles using bright-field transmission electron microscopy (BF-TEM) and thus represents a lower-bound estimate.^44^ Oleylamine-capped Au nanospheres were obtained following a modified burst nucleation method of Peng et al.,^45,46^ yielding nanoparticles with an average diameter of 3.5 ± 0.4 nm, thereby matching the nanorod thickness. The interparticle surface-to-surface distance, determined from BF-TEM images, was 1.9 ± 0.4 nm, which corresponds to typical surface-to-surface spacings for oleylamine-stabilized Au nanoparticles.^46,47^

**Figure 1:**
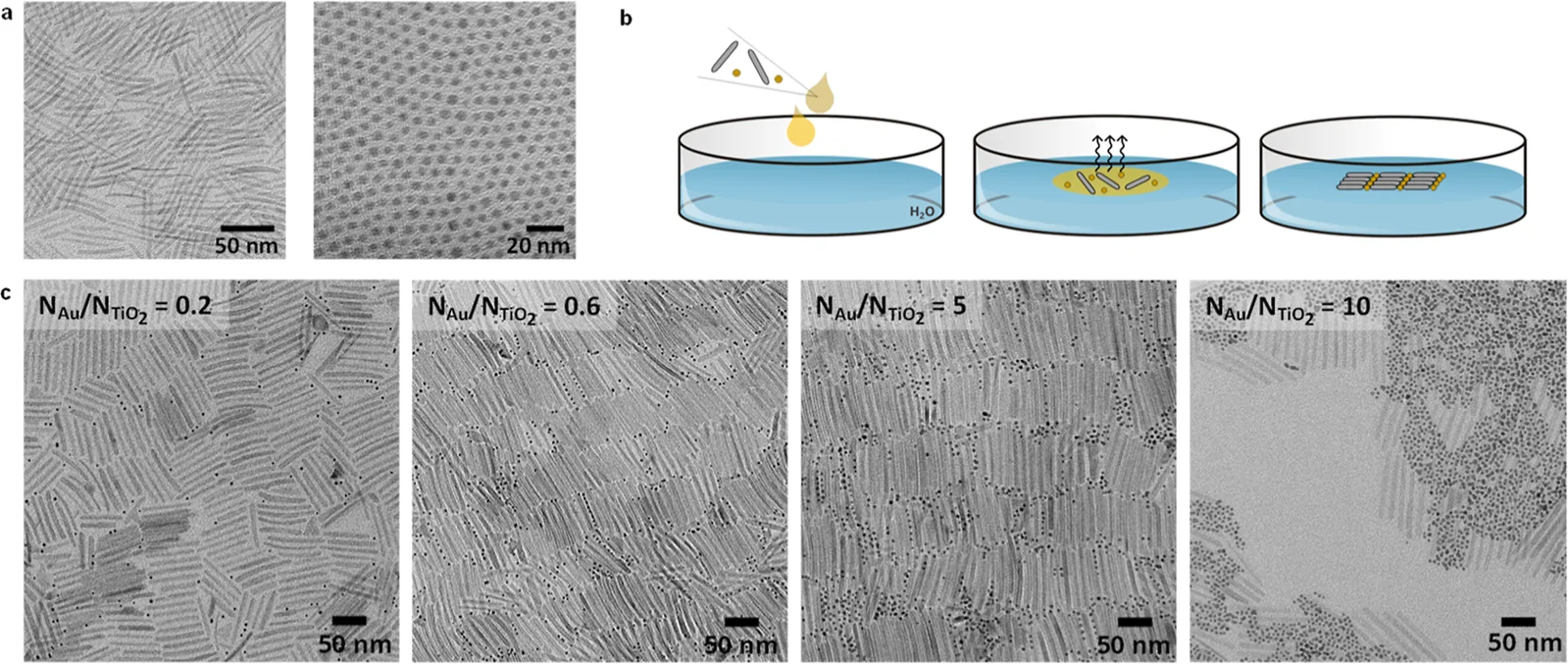
2D self-assembly of TiO_2_ nanorods and Au nanospheres. (a) BF-TEM images of as-synthesized TiO_2_ nanorods (49 ± 7 nm; 3.5 ± 0.6 nm) and Au nanospheres (3.5 ± 0.4 nm). (b) Schematic illustration of Au/TiO_2_ binary superlattice formation by evaporation-driven assembly of a nanoparticle dispersion in oil (yellow) at a water–air interface. (c) BF-TEM images of the resulting Au/TiO_2_ superlattices with increasing Au-to-TiO_2_ particle number ratios (*N*_Au_/*N*_TiO2_) from left to right.

The phase behavior of Au nanospheres and TiO_2_ nanorods was first studied using a 2D assembly approach.^24,43,48^ Here, an apolar Au/TiO_2_ sphere–rod dispersion in toluene was allowed to slowly evaporate on a polar water–air interface (Figure 1b), as described in Experimental Methods. The resulting Au/TiO_2_ film was transferred onto a TEM grid for analysis. BF-TEM images in Figure 1c show that well-defined layers of TiO_2_ nanorods were formed with Au nanospheres positioned at the nanorod tips. With increasing Au concentration, indicated by particle number ratio (*N*_Au_/*N*_TiO2_), multiple layers of Au nanospheres were observed in between single nanorod layers. This is most evident for a *N*_Au_/*N*_TiO2_ of 5 in Figure 1c. The Au nanospheres were placed not only at the tips of the nanorods but also along the rod length. This resulted in substantial disruption and isolation of TiO_2_ rod layers for a *N*_Au_/*N*_TiO2_ between 5 and 10, indicating a membrane phase rather than a binary smectic phase. For a *N*_Au_/*N*_TiO2_ of 10, the rods and spheres did no longer mix, likely due to depletion interactions. This is also indicated by the voids in the film that suggest that the system has fallen out of equilibrium. Therefore, an optimal particle ratio for achieving smectic rod alignment was determined to be around 5 or lower.

### The Formation of Binary Supraparticles through 3D Assembly

Using the optimal particle number ratios from 2D assembly, Au/TiO_2_ supraparticles were formed through a 3D evaporation-induced assembly approach (Figure 2a). This method was based on de Nijs et al.,^38^ where oil-in-water emulsion droplets were slowly dried, leading to close packing of nanoparticles in spherical supraparticles. In this work, the oil phase consisted of a dispersion of Au nanospheres and TiO_2_ nanorods in toluene, emulsified in an aqueous continuous phase, containing SDS to stabilize the emulsion droplets (see the Experimental Methods).

**Figure 2:**
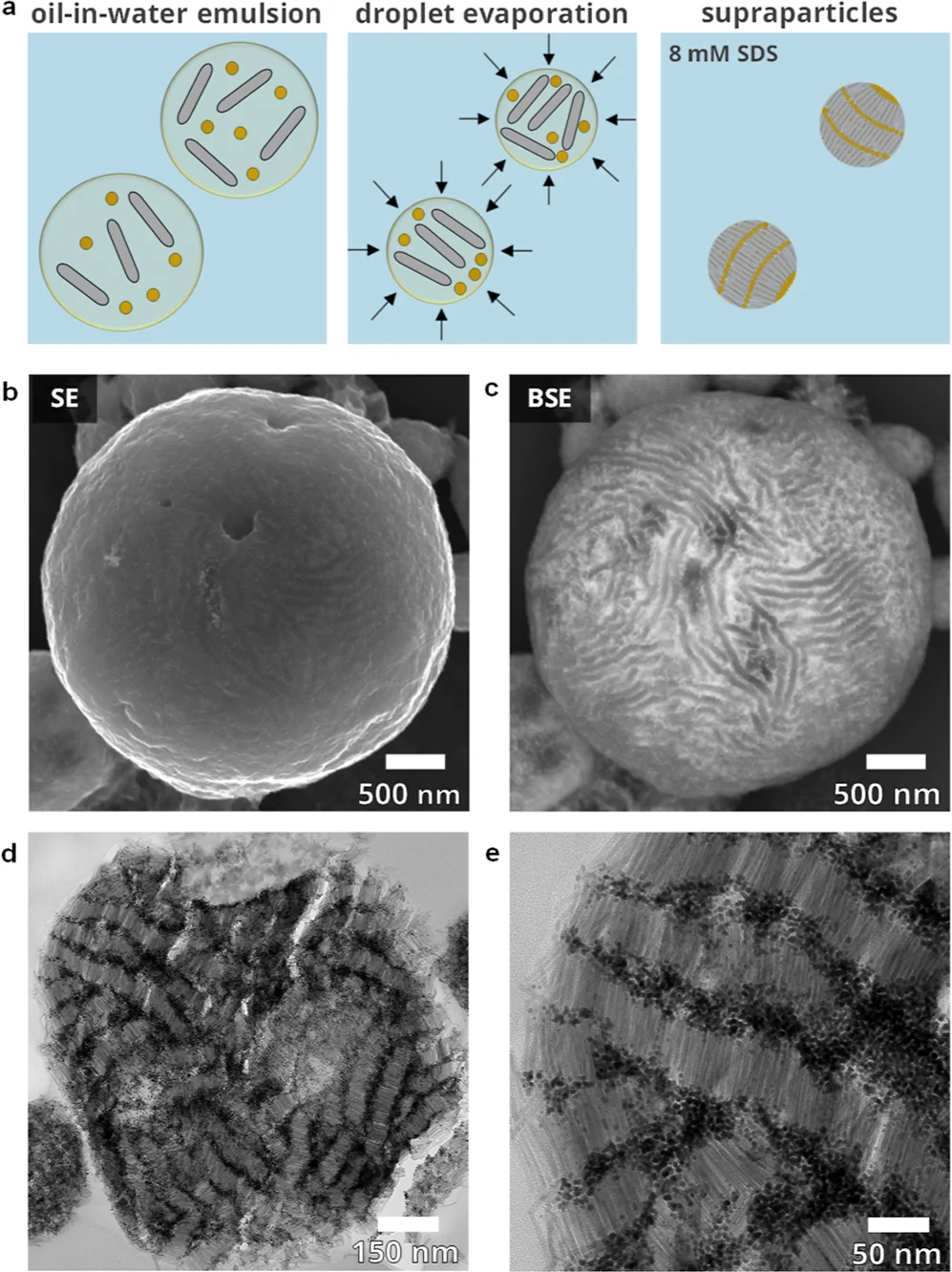
3D self-assembly of TiO_2_ nanorods and Au nanospheres into membrane supraparticles with a *N*_Au_/*N*_TiO2_ of 5. (a) A schematic illustration of the evaporation-driven assembly of oil-in-water emulsion droplets containing a binary Au/TiO_2_ mixture into supraparticles. (b, c) Scanning electron microscopy (SEM) images of a membrane supraparticle acquired with secondary electrons (b) and backscattered electrons (c). (d, e) BF-TEM images of an ultramicrotome section of a supraparticle at low (d) and high (e) magnification.

Binary supraparticles were prepared with *N*_Au_/*N*_TiO2_ values of 0.05, 0.6, and 5. The Au weight loading was determined by energy dispersive X-ray (EDX) analysis to be 1.1 ± 0.2 wt·%, 10.1 ± 1.3 wt·%, and 40.6 ± 3.2 wt·%, respectively. These experimental Au loadings are in good agreement with theoretical calculations (Table 1), detailed in Supporting Information 2.2.

**Table 1:** Quantification of the Au Loading in the Binary Supraparticles by EDX Measurements Compared to the Expected Au Weight Loading

particle number ratio [*N*_Au_/*N*_TiO2_]	expected Au loading (wt·%)	experimentally measured Au loading (wt·%)
5	45.7	40.6
0.6	9.7	10.1
0.05	1.1	1.1

Figure 2b,c shows SEM images of a resulting Au/TiO_2_ supraparticle with a *N*_Au_/*N*_TiO2_ of 5. The SE image in Figure 2b shows a spherical supraparticle of approximately 2.4 μm. Taking the previously determined interparticle spacings into account, the numbers of nanorods and nanospheres in this supraparticle were estimated to be around 3.8 × 10^6^ and 1.5 × 10^7^ particles, respectively. The supraparticle contained several pores, which were likely caused by wetting of the oil phase, resulting in water droplets inside the oil-in-water emulsion droplets.^49,50^ The backscattered electron (BSE) image in Figure 2c reveals the distribution of the TiO_2_ and Au nanoparticles caused by differences in Z-contrast. The observed dark stripes represent TiO_2_ nanorod layers, while the Au nanospheres in between appear bright due to the higher atomic number of Au. This nanoparticle distribution observed by contrast variation in Figure 2c shows the presence of TiO_2_ rod layers isolated with thick layers of Au nanospheres, indicating the formation of colloidal membranes.

To study the ordering inside the supraparticles, we employed ultramicrotomy. Here, supraparticles were embedded inside a resin, which was subsequently cut into thin slices (50–70 nm) with a diamond knife. Figure 2d,e shows BF-TEM images of a ultramicrotomy sectioned Au/TiO_2_ supraparticle at low and high magnification. Note that the Au nanoparticles appear dark in the BF-TEM images in Figure 2d,e, whereas they appear bright in the BSE-SEM image in Figure 2c. The BF-TEM images confirm that multiple Au sphere layers were formed in between single TiO_2_ rod layers, which is characteristic for a phase composed of colloidal membranes.^31,32^ Since membranes were observed both inside the supraparticle (Figure 2d,e) and on the surface (Figure 2b,c), colloidal membranes were likely formed throughout the entire supraparticle.

By adjusting *N*_Au_/*N*_TiO2_, we achieved binary smectic ordering in the 3D assemblies. Figure 3 shows the structural characterization of Au/TiO_2_ supraparticles with lower Au sphere concentrations: *N*_Au_/*N*_TiO2_ = 0.6 and 0.05. The assembly of only TiO_2_ nanorods into superlattices and supraparticles is shown in Figure S1. The SE-SEM images in Figure 3a,e show that the supraparticles have a similar morphology as for a *N*_Au_/*N*_TiO2_ of 5 in Figure 2b. However, the elemental contrast provided by backscattered electrons in Figure 3b,f decreased with decreasing Au content. For a *N*_Au_/*N*_TiO2_ of 0.05, the Au signal was too weak to distinguish the nanospheres from the TiO_2_ nanorods. However, high-angle annular dark-field scanning transmission electron microscopy (HAADF-STEM) images with corresponding EDX elemental maps (Figures 3d,h and S2) confirm the presence of Au nanospheres in between the TiO_2_ nanorod layers.

**Figure 3:**
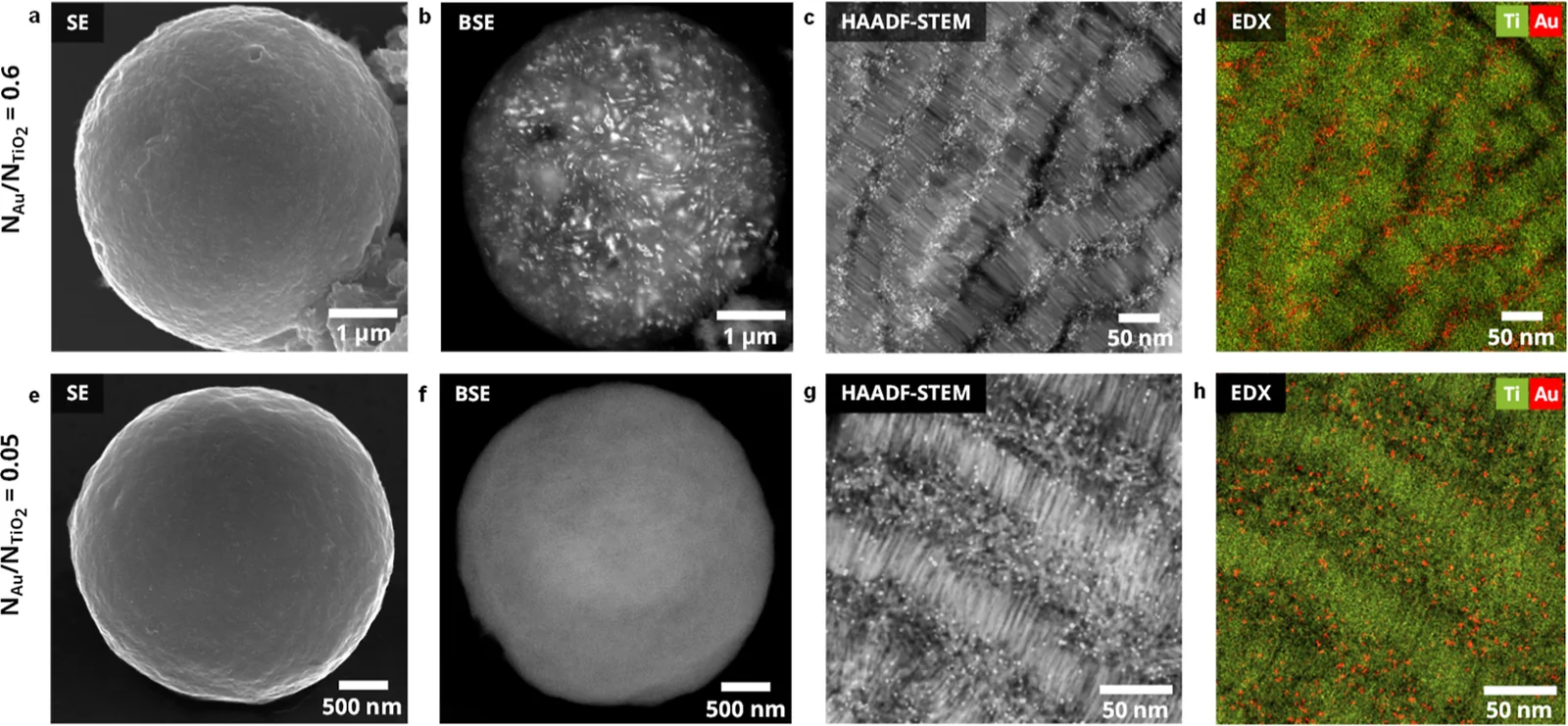
Structural characterization of Au/TiO_2_ supraparticles with *N*_Au_/*N*_TiO2_ values of 0.6 and 0.05. (a, b, e, f) SEM images of a representative Au/TiO_2_ supraparticle captured with SE and backscattered electron (BSE) detectors. (c, d, g, h) HAADF-STEM images of ultramicrotomy sections of Au/TiO_2_ supraparticles with corresponding EDX elemental map, showing the nanoparticle distribution of Au (red) and Ti (green).

For a *N*_Au_/*N*_TiO2_ of 0.6, the number of layers of spheres in between the TiO_2_ layers was substantially reduced (Figure 3c,d) compared to the membrane supraparticles with a *N*_Au_/*N*_TiO2_ of 5 in Figure 2. Using HAADF-STEM tomography on an ultramicrotomy sectioned supraparticle, we achieved 3D analysis of a sufficiently thin cross-section of a binary supraparticle to permit electron transmission and show that it has a binary smectic structure (Figure 4). A tilt series was performed from −59° to 75° of which a selection of angles is shown in Figure 4a. The full tilt series is shown in Movie S1. The 3D reconstructed model in Figure 4c shows the presence of smectic TiO_2_ layers alternating with thin layers of Au spheres, characteristic for binary smectic ordering. The voids observed in the 3D model in Figure 4c are likely artifacts arising from the reconstruction of the thin nanorods and additionally from the ultramicrotomy procedure. The reconstructed model is shown in 3D in Supporting Information—3D reconstruction.

**Figure 4:**
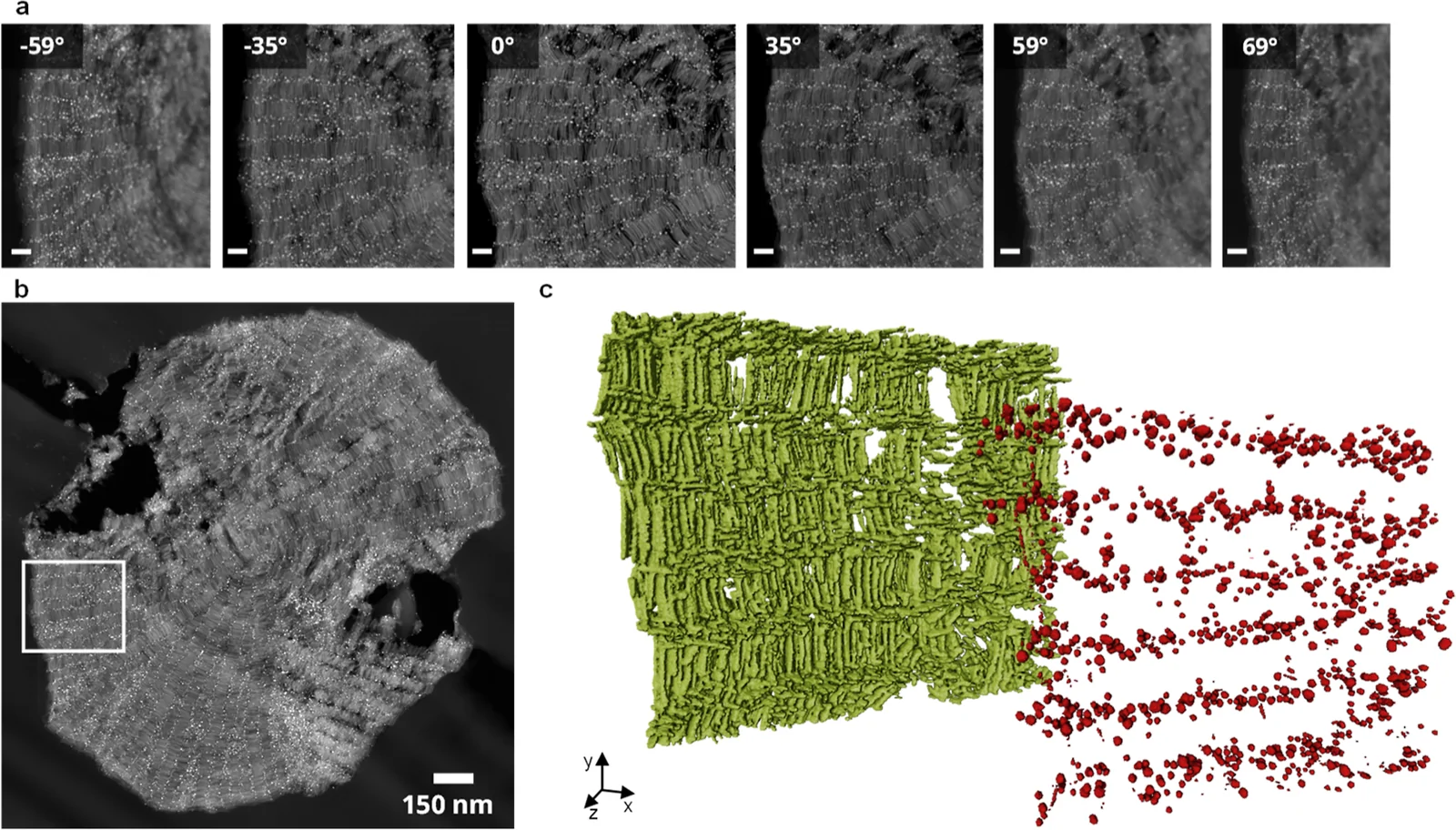
3D analysis of a Au/TiO_2_ supraparticle with a *N*_Au_/*N*_TiO2_ of 0.6 using HAADF-STEM tomography. a) An ultramicrotomy section of a supraparticle over a tilt range from −59° to +69° with scale bars of 50 nm. b) Overview of the entire ultramicrotomy section of the supraparticle. c) Corresponding 3D reconstruction of individual nanorods in green and spheres in red.

Interestingly, further decreasing the Au content to a *N*_Au_/*N*_TiO2_ of 0.05 did not result in alternating smectic rod layers with a lower number of Au spheres in between. In fact, TiO_2_ nanorods were also detected in between the smectic layers with EDX (Figures 3h and S2). This is also suggested by the larger distance between the smectic layers of *N*_Au_/*N*_TiO2_ = 0.05 compared to *N*_Au_/*N*_TiO2_ = 0.6, indicating that the smectic gap was not exclusively filled with Au nanoparticles (Figure S2). As smectic ordering can occur in multiple directions, we also performed a 3D analysis with HAADF-STEM tomography on this sample to resolve the nanoparticle arrangement. A tilt series was performed from −66° to 66° of which a selection of angles is shown in Figure S3. The reconstructions in Figure S3b–d confirm that the interstitial space between smectic TiO_2_ layers was filled up with disordered TiO_2_ nanorods mixed with Au nanospheres. We argue that this could be caused by an insufficient concentration of spheres to overcome the entropy of mixing,^24^ or by kinetic arrest, which traps the assembly process in a nonequilibrium state.

### Monitoring Binary Smectic Ordering in Supraparticle Formation

The evaporation of emulsion droplets into supraparticles was followed over time using POM (Figure 5). In contrast to electron microscopy techniques, optical microscopy enables in situ characterization, allowing us real-time monitoring of evaporating emulsion droplets in which nanoparticle assembly occurs. In addition, polarized light was used to assess the degree of ordering within supraparticles over large sample areas.^40^ When the supraparticles are disordered, they behave as an isotropic material that does not alter the polarization of light. In contrast, membrane and binary smectic ordered supraparticles exhibit birefringence (i.e., difference in refractive indices parallel and perpendicular to the director), which can modify the light’s polarization state.^51^ In the latter case, even with the analyzer oriented at 90° to the polarizer, a component of the light can pass through the detector, resulting in bright signals.

1 $$I_{⊥}=I_{0}\sin^{2}\left(2\theta \right)\sin^{2}\left(\frac{\pi d\Delta n}{\lambda }\right)$$

**Figure 5:**
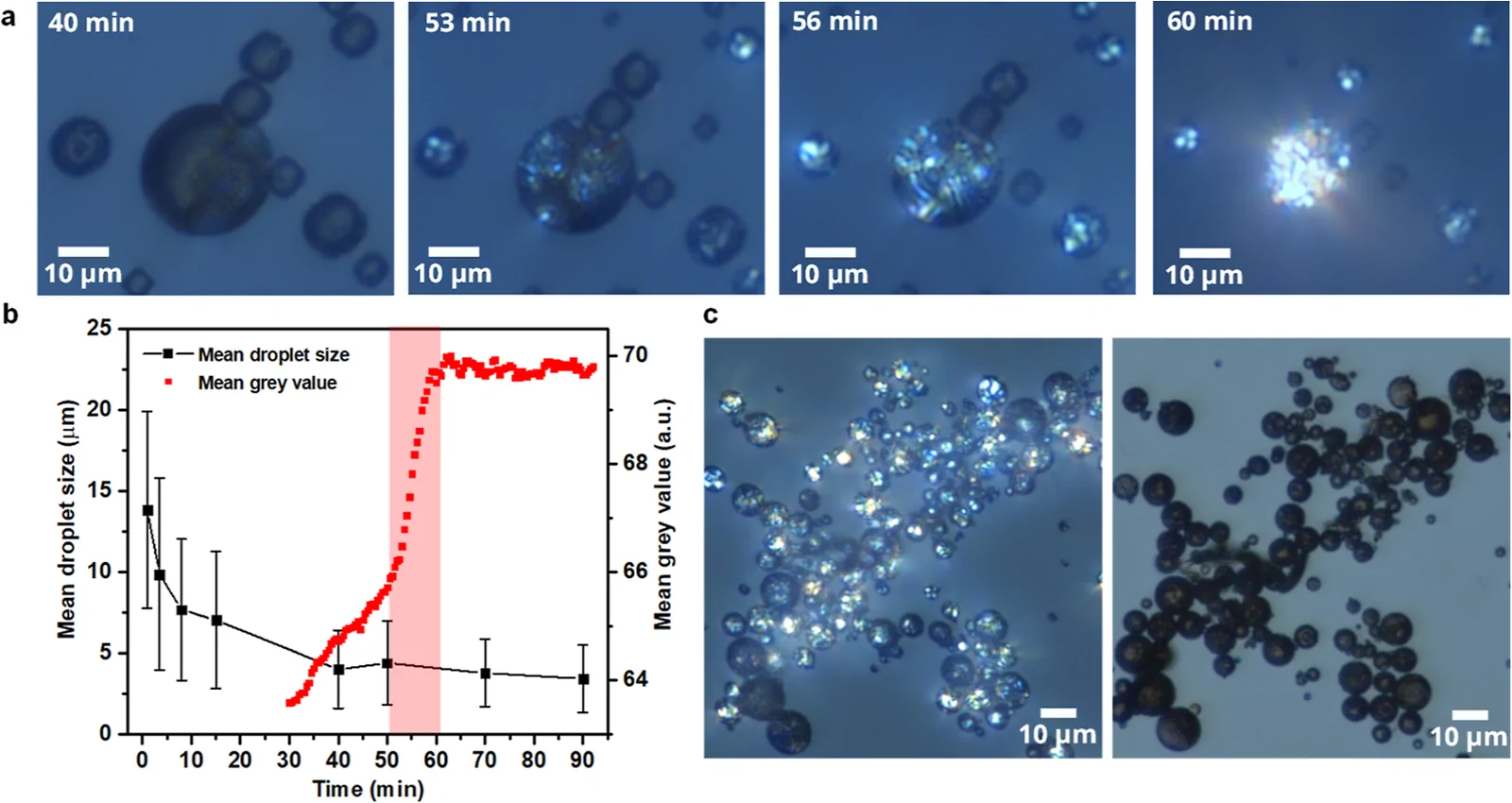
Monitoring the formation of Au/TiO_2_ supraparticles (*N*_Au_/*N*_TiO2_ = 0.05) over time using POM. (a) Time-lapse cross-polarized optical microscopy images of emulsion droplet evaporation into supraparticles. (b) The decrease in emulsion droplet size (*n* = 300) over time compared to the increase in gray value. A steep increase in gray value is highlighted in red, showing the development of smectic ordering. (c) A cross-polarized (left) and corresponding bright-field (right) light microscopy image of the resulting supraparticles.

The light intensity from transmitted polarized light between crossed (*I*_⊥_) is described by eq 1 where *I*_0_ represents the incoming light intensity, *d* is the sample thickness, θ is the angle between the polarizer and the optical axis, λ is the wavelength, and Δ*n* is the refractive index difference (birefringence).^52–54^

To allow for real-time observation of the supraparticle formation process, the assembly was carried out in a covered Petri dish instead of the typically used covered glass vial (see the Experimental Methods). This, in turn, accelerated the assembly from half a day to approximately an hour. Since the drying time can affect ordering, estimations on the degree of ordering were based on supraparticles assembled ex situ in a covered glass vial.

Figure 5a shows the evaporation of emulsion droplets (*N*_Au_/*N*_TiO2_ = 0.05) over time under cross-polarizers. Using the cross-polarized images, it was determined that the average emulsion droplet size (*n* = 300) decreased from 14 ± 6 μm to 3 ± 2 μm over 90 min with the largest decrease within the first 10 min. Note that differently sized emulsion droplets shrink with different rates, resulting in a relatively large polydispersity in droplet size, particularly in the early stage of evaporation. Figure 5b shows that the mean droplet size remained constant between 70 and 90 min, indicating that the evaporation of the emulsion droplets was completed by 70–90 min. During the evaporation of emulsion droplets, we did not observe any droplets coalescing or breaking up. The light intensity of the cross-polarized microscopy images was analyzed by converting the RGB images to 8-bit grayscale from which the mean gray value for each image was calculated (detailed in Supporting Information 1.2). As shown in Figure 5b, the resulting mean gray values, representing the overall brightness of each microscopy image, increased over time. After approximately 50 min, when the droplets had shrunk 77% on average, a steep increase in mean gray value was detected (Figure 5b). This was due to supraparticles becoming birefringent upon the formation of a locally aligned liquid crystal phase (Figure 5a). The gradual increase in gray value from 30 to 50 min is likely caused by the shrinkage of the emulsion droplets, resulting in higher fraction of the lighter background. Light scattering can also contribute to the increase in light intensity. However, the observed increase in light intensity in Figure 4b cannot be attributed solely to scattering of light. Larger emulsion droplets generally scatter more light; therefore, if the increase in brightness were caused only by scattering, the light intensity is expected to decrease during evaporation, which is a trend we do not observe (Figure 4b). Furthermore, some smaller supraparticles appear brighter than some larger supraparticles under cross-polarizers in Figure 5c, which cannot be explained by scattering effects alone.

By monitoring the supraparticle formation in situ, we can also determine the volume percentage occupied by nanoparticles relative to the total droplet volume over time. This can be used to deduce the impact of attractive interactions on the assembly. The volume percentage of particles when the birefringence response was first recorded was found to be 24% ± 2%, calculated by relating the decrease in droplet size to the initial droplet size, given an initial particle volume percentage of 0.75%. The initial droplet size had to be estimated because evaporation caused significant movement within the first minutes and due to sedimentation of the droplets, becoming denser during the evaporation into supraparticles. This particle volume fraction was slightly lower than that for TiO_2_ supraparticles for which the birefringence was first measured at a volume percentage of 28% ± 2%. This suggests not only that the presence of Au spheres induces additional attractive interactions, promoting smectic ordering at lower volume fractions, but also that the effects of the attractions were not substantial.

The degree of ordering was estimated from the birefringence response of the supraparticles under crossed polarizers. By comparing bright-field and corresponding cross-polarized light microscopy images of ex situ assembled supraparticles, it was estimated that approximately 87% of supraparticles (*n* = 242) appeared bright under cross-polarizers, indicating anisotropic phase behavior (Figure S4). The rest of the supraparticles remained dark under cross-polarizers, indicating that 13% of the supraparticles behaved as an isotropic material and therefore exhibited disorder. However, it should be noted that the light intensity depends on the sample thickness (eq 1), so the smallest supraparticles may not be sufficiently dense to produce a detectable signal. Also, the light intensity of transmitted polarized light is dependent on the orientation of the optical axis relative to the polarizers (eq 1). This can result in birefringent supraparticles being undetected due to an unfavorable orientation.

Supraparticles with higher Au concentrations were also analyzed with POM (Figure S5). For a *N*_Au_/*N*_TiO2_ of 0.6, the birefringence response was similar to a *N*_Au_/*N*_TiO2_ of 0.05. However, the supraparticles appeared red under cross-polarizers due to the increasing Au concentration. For a *N*_Au_/*N*_TiO2_ of 5, the birefringence response was minimal. This suggests that only a fraction of supraparticles exhibited membrane-like ordering. However, we argue that the signal could also be diminished by the absorption of visible light by plasmonic Au particles. As a result, the degree of ordering corresponding to the birefringence response can be estimated for supraparticles with no (Figure S6) or low Au concentrations, whereas higher Au loadings (≥10 wt·%) might lower the light intensity reaching the detector.

#### Determining the Bulk Phase Diagram for Rod–Sphere Mixtures Using MD Simulations

To gain insight into the equilibrium phase behavior and phase transformation kinetics, we performed MD simulations of a binary mixture of hard spheres with diameter σ and hard rods with an aspect of *L*/σ = 10 in the *NPT* ensemble, where we fix the number of rods and spheres, the pressure, and the temperature. We mapped out the phase diagram of the rod–sphere mixture as shown in Figure 6. The system is initialized with a regular spatial distribution of particles, see Figure S7. Rods were represented as rigid bodies composed of ten nonoverlapping spheres of diameter σ. However, the packing fraction was calculated using the equivalent spherocylinder volume, yielding a rod volume *V*_rod_ = 31πσ^3^/12. We initialized the simulations from a regular array of rods, as shown in Figure S7, and varied the sphere/rod ratio by replacing a fraction of the rods with spheres. The system was then compressed to the desired pressure and equilibrated for 4.5 × 10^5^ iterations. We subsequently calculated time-averaged packing fractions and nematic and smectic order parameters over the final 10 snapshots, sampled at intervals of 5 × 10^4^ iterations. We performed a parameter sweep over particle number ratios and packing fractions and characterized the resulting phase behavior using the nematic and smectic order parameters. The nematic order parameter *S* is defined as the largest eigenvalue of the nematic order parameter tensor

2 $$Q_{\alpha \beta }=\frac{1}{N_{rod}}\sum_{i=1}^{N_{rod}}\left[\frac{3}{2}u_{i\alpha }u_{i\beta }-\frac{\delta_{\alpha \beta }}{2}\right]$$

where u_*i*α_ is the α-th component of the unit vector describing the orientation of the long axis of rod *i*, *N*_rod_ is the number of rods, and δ_αβ_ is the Kronecker delta (eq 2). The eigenvector corresponding to the largest eigenvalue defines the nematic director **n**. The nematic order parameter varies between −0.5 and 1, corresponding to transverse alignment and perfect alignment, respectively. The smectic order parameter we used is defined as

$$\tau =\underset{l}{\max}\left|\overset{N_{rod}}{\underset{j=1}{\sum }}e^{2\pi ir_{j}\cdot n/l}\right|$$ 3

where the value of l∈R that maximizes the above expression is identified as the layer spacing *d* (eq 3). Similar to, τ is bounded between −0.5 and 1, corresponding to irregular or absence of layering and perfectly ordered layers, respectively. We classified the observed phases as follows: isotropic phases exhibit low nematic order, *S* < 0.5; nematic phases show high nematic but low smectic order, S ≥ 0.5, τ < 0.35; and smectic phases display both high nematic and smectic order, S ≥ 0.5, τ ≥ 0.35. These threshold values influence the identification of the different phases and were chosen based on previous work^26^ and visual inspection.

**Figure 6:**
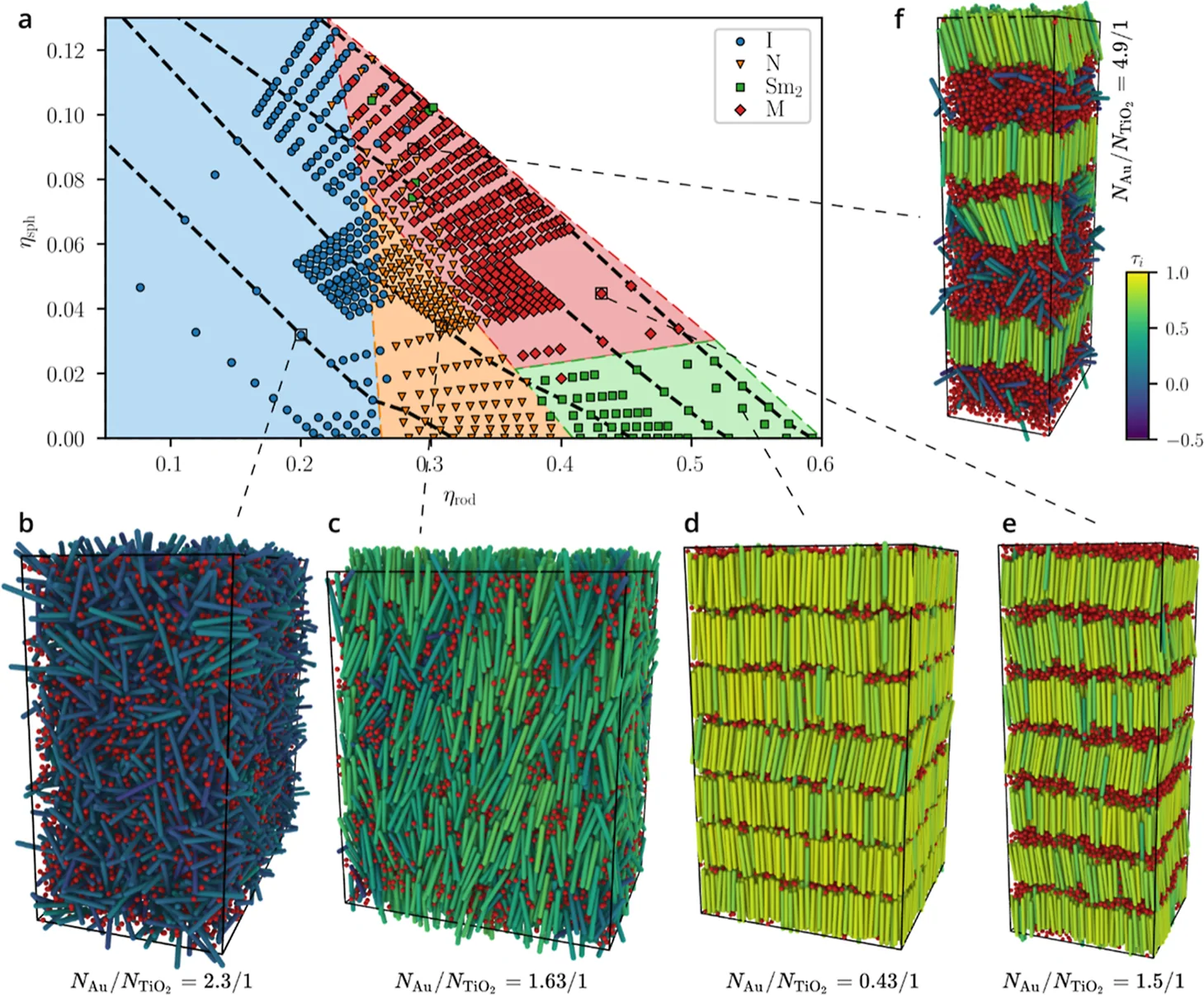
Phase diagram obtained from MD simulations as a function of the packing fraction of rods η_rod_ and spheres η_sph_. Blue circles indicate the isotropic phase (I), orange triangles the nematic phase (N), green squares the binary smectic phase (Sm_2_), and red diamonds the colloidal membrane phase (M). The dashed lines correspond to the pressure isotherms of β*P*σ^3^ = [0.4, 0.8, 1.2, 1.6]. Representative snapshots are shown for (b) isotropic, (c) nematic, (d) binary smectic, and (e, f) membrane phases. Spheres are colored red, while rods are colored according to their local smectic order parameter τ_*i*_ (see Experimental Methods).

In the phase diagram, blue circles represent isotropic phases (Figure 6b), orange triangles denote nematic phases (Figure 6c), green squares indicate smectic phases (Figure 6d), and red diamonds correspond to colloidal membrane phases (Figure 6e,f). In the representative snapshots, Figure 6b–f and Movie S2, spheres are colored red, while rods are colored according to their local smectic order parameter τ_*i*_ (see Experimental Methods). Colloidal membrane phases correspond to systems in which monolayers of rods are surrounded by spheres. Although these systems exhibit high nematic and smectic order, they are distinguished by a larger layer spacing *d* > *L*+2σ, resulting in weak or no correlations between adjacent layers.

We observed that at low sphere packing fractions, the phase transitions are accompanied by a discontinuity in the rod packing fraction. This behavior is expected as increasing nematic order leads to more compact configurations. Furthermore, the presence of spheres has a negligible effect on the isotropic–nematic transition, as made evident by the nearly vertical boundary of the isotropic region. In contrast, the addition of spheres lowers the packing fraction at which the nematic–smectic transition occurs. This suggests that depletion interactions induced by the presence of spheres favor the formation of smectic layers. This interpretation is consistent with experimental observations, where high sphere concentrations lead to layered supraparticles with high smectic order (Figure 3). Despite starting from an initially homogeneous spatial distribution of particles, we find that the binary smectic phase nucleates from the isotropic phase upon quenching the system to high pressure, see Movie S3. We observe that the nucleation pathway of the binary smectic phase proceeds through the formation of single layers of rods, which subsequently assemble into larger clusters as successive smectic layers nucleate and grow on top of the existing layers. This pathway is consistent with previous work regarding the nucleation of a smectic phase in systems of pure hard rods.^27^

At high sphere packing fractions, the nematic and smectic phases are no longer observed and the system instead undergoes a direct transition from the isotropic to the membrane phase. The membrane phase self-assembles at sufficiently high sphere–rod particle number ratios, above *N*_Au_/*N*_TiO2_ of 1, as shown in Figure S8. At these particle number ratios, spheres located between the membranes disturb the ordering of a subset of rods, as evident in Figure 6f and in the one-dimensional composition profiles shown in Figure S9. As a result, only a fraction of the rods participate in well-defined layers, which may account for the reduced birefringence observed at *N*_Au_/*N*_TiO2_ = 5 (Figure S4). The colloidal membranes are long-lived and significantly hinder the diffusion of spheres across the rod layers, as shown by simulations of an initially unmixed system along the direction of the rod main axes, see Supplementary Movie S4. Despite the restricted diffusion of spheres across rod layers, spheres can diffuse freely within interlayer regions. Consequently, an initially unmixed system in the direction transverse to the rod main axes evolves toward the membrane phase, as shown in Supplementary Movie S5.

## Discussion

The Au/TiO_2_ supraparticles presented in this paper exhibited nanoparticle ordering in alternating TiO_2_ rod and Au sphere layers, forming colloidal membrane or binary smectic phases. The alignment of the nanorods is likely driven by minimization of excluded volume, as described by Onsager’s theory,^20^ where rod alignment enhances translational entropy sufficiently to compensate for the loss of orientational entropy. In the presence of Au nanospheres, the interstitial space between the rod domains were occupied by spheres, likely supported by depletion-driven entropic interactions.^24,55^ In this study, we focused on a system in which the interactions were dominated by hard-particle interactions. This is important, as self-assembly governed by additional interactions would be less general (e.g., attractions often depend on particle size). This is particularly relevant for binary systems, where excluded-volume interactions are already sufficiently complex. We have experimentally promoted such a hard-particle system by post-treating the rods to reduce attractive interactions.^43^

We found that the particle number ratio in the sphere–rod mixture plays a key role in determining the type of ordering. This is consistent with the work of Ye et al. on binary 2D superlattices, where the concentration of spheres must be sufficiently high to form single layers of spheres that alternate with rod layers, thereby competing with the entropy of mixing.^24^ Additionally, Lei et al. found that low sphere contents favored rod–rod contact and suppressed binary ordering, while increasing the sphere concentration promoted rod–sphere coassembly.^56^ Similarly, we found that a relatively high sphere concentration (*N*_Au_/*N*_TiO2_ = 0.6) is required to stabilize the binary smectic phase (Figure 4), whereas at lower sphere concentrations (*N*_Au_/*N*_TiO2_ = 0.05), the Au spheres remain mixed with TiO_2_ rods in between smectic TiO_2_ layers (Figure 3b). Ye et al. observed that the sphere concentration can also be too high, resulting in bulk phase separation.^24^ In addition, Bakker et al. showed that stable binary smectic phases occur only for fractions of spheres (*x*_sph_ = *N*_sph_/*N*) lower than 0.8.^26^ Our self-assembly experiments on a water–air interface with Au sphere–TiO_2_ rod mixtures are consistent with these findings, as we found that for *N*_Au_/*N*_TiO2_ above 5, corresponding to *x*_sph_ > 0.83, smectic ordering is strongly disrupted (Figure 2). In contrast, our 3D supraparticles exhibited colloidal membranes at a high sphere concentration (Figure 3a). This observation is corroborated by our simulations, which show membrane formation at *N*_Au_/*N*_TiO2_ = 5. Experimentally, we find that even at extremely high particle number ratios (*N*_Au_/*N*_TiO2_ of 6–38), the rods can still align into (single) layers (Figure S10), suggesting that complete segregation is unlikely. Although this is in contrast with our simulations, which predict isotropic phase behavior for *N*_Au_/*N*_TiO2_ values of 13, 25, and 38 (Figure S8), we argue that this discrepancy is likely caused by finite-size effects in the simulations. We believe that the small density differences between the phases and the high viscosity of the suspension prevented macrophase separation driven by sedimentation within the assembly time frame.

To gain a better understanding of the transition from the binary smectic phase at *N*_Au_/*N*_TiO2_ = 0.6 to the membrane phase at *N*_Au_/*N*_TiO2_ = 5, additional structural characterization of Au/TiO_2_ supraparticles with an intermediate particle number ratio was performed (*N*_Au_/*N*_TiO2_ of 2.5), as shown in Figure S11. Although a gradual transition from binary smectic to membrane phase upon increasing the Au content cannot be concluded based on experimental data alone, the gradual increase in layer spacing found with numerical simulations (Figure S9) suggests that such a gradual phase transition may be possible.

In addition to the sphere-to-rod particle number ratio, the rod aspect ratio appears to influence the stability of the binary assemblies. Lei et al. reported that the formation of their ordered binary assemblies was restricted to only narrow regions of their phase diagram,^56^ whereas we observe relatively broad regions in which the binary smectic and membrane phases were stable. We argue that this difference is related to the aspect ratios of the rods used. Kuijk et al. previously reported an aspect ratio of 4.1 as a threshold for the formation of smectic liquid crystallinity.^30^ While Lei et al. used rods with an aspect ratio below 4, our rods had an aspect ratio of approximately 8, which may favor the formation and stability of ordered binary assemblies over a much broader parameter range.

Our study further shows that POM measurements can be used for in situ analysis of supraparticle formation, complementing electron microscopy techniques, while simultaneously providing information on the ordering of supraparticles. The anisotropic phase behavior started around a volume percentage of 28% ± 2% for TiO_2_ supraparticles. Consistent with this observation, our numerical simulations (Figure 6a) show that hard rods undergo a transition from an isotropic to a liquid crystalline phase at a similar volume fraction. Although attractive interactions next to depletion effects are likely present in our system, this strongly suggests that the presence of these attractive interactions do not dominate the phase behavior substantially.

Although this study demonstrated the successful formation of supraparticles exhibiting binary smectic and colloidal membrane phases, the binary assembly can be further optimized.^26^ Pits were observed in the supraparticles, which could arise due to wetting of the oil phase and/or by (swollen) micelles that get adsorbed into the supraparticles.^49,50^ While this porosity can be advantageous for catalytic applications,^57^ the pores can probably be avoided by further tuning the oil-to-water ratio or the amount of excess surfactant present in the continuous phase.^50^ Interestingly, the pores observed in Figures 2 and S1c did not seem to induce disorder. Other inhomogeneities, such as the supraparticle size, can be controlled by using microfluidics. This technique should then be combined with methodologies that allow for synthesis scale-up.^58–60^

Our rod–sphere assemblies typically consist of curved rod layers. This curvature is most apparent at high *N*_Au_/*N*_TiO2_ where membrane phase is formed (Figure 2). This is not surprising, as membranes are typically described as fluid-like and elastic structures that can undergo significant fluctuations, as studied by the Dogic group.^31,32^ However, some degree of curvature is also observed in both the 2D assemblies (Figures 1c and 3b–d) and supraparticles at lower *N*_Au_/*N*_TiO2_, indicating that curvature is not exclusive to the membrane phase. Additionally, differently oriented rod domains can be observed (Figures 4b and S3). Wang et al. attribute the formation of these domains to a so-called ‘embryo-crystallization process’ in which small ordered regions nucleate independently and grow into layers while adopting distinct rod orientation.^61^ This mechanism appears to be consistent with our simulations on the nucleation of a binary smectic phase, as shown in Supplementary Video S3.

The rod–sphere assembly into binary smectic and membrane phases resulted in sphere-at-rod-tip ordering. Previous studies on rod–sphere assemblies have reported different types of ordering, such as side-by-side arrangement. For example, Nagaoka et al. introduced additives to promote attractive sphere–rod interactions, driving the spheres to intercalate within the rod arrays rather than phase separating during assembly.^62^ Similarly, Sánchez-Iglesias et al. used ultralong nanowires (>5 μm) to direct the spheres to the sides of the wires.^63^ Notably, both papers introduced additional components to achieve binary assembly (in 2D), resulting in side-by-side sphere–rod assemblies. In contrast, our work does not add such components and achieves sphere-at-rod-tip ordering. As colloidal membranes and, in particular, binary smectic ordering control the position of the spheres at the tips of the rods, this nanoparticle arrangement offers a promising strategy for enhancing the synergistic effects of tip-localized properties. This in combination with supraparticle assembly makes them interesting for a variety of applications. The presented Au/TiO_2_ supraparticles are interesting for photocatalytic applications. Au-supported TiO_2_ is widely recognized as promising photocatalysts due to their ability to absorb a broad range of the solar spectrum.^39^ Using TiO_2_ in the form of nanorods has already been demonstrated to improve the separation of photogenerated electron–hole pairs.^42^ Placing the Au nanospheres next to the nanorod tips through binary smectic ordering could make electron transfer more efficient, thereby further suppressing charge recombination. Combining this with the enhanced light–matter interactions arising from their assembly into supraparticles^35,37^ could yield highly active photocatalysts. Moreover, the binary smectic arrangement may help limit nanoparticle sintering, as the spheres are partially surrounded by rods, potentially improving catalytic stability.^64^

Binary smectic and membrane supraparticles are not limited to Au nanospheres and TiO_2_ nanorods, but various combinations of rod–sphere building blocks can be used. For instance, ordered supraparticles composed of quantum dots and semiconductor nanowires, such as CdSe/ZnO, can be promising solar cells for similar reasons, namely, improved light–matter interaction and charge separation.^65^ Supraparticles containing rods and spheres also hold potential for energy storage. For example, Xue et al. reported that Fe_3_O_4_-doped TiO_2_ nanorod supraparticles exhibited enhanced lithium storage performance.^66^ Rod–sphere alignment would likely further improve electron transport and Li^+^ diffusion, resulting in an additional enhancement in battery performance. Another application can be found in sensors, as semiconductor nanowires loaded with noble metals can increase sensor sensitivity and selectivity.^67^ Smectic ordering could enhance local electronic responses upon gas exposure, improving the sensor signals. The ordered, multicomponent nature of binary smectic or membrane supraparticles also makes them attractive in optical applications, e.g., by using local electromagnetic field enhancement at nanorod tips, which strongly amplifies the surface-enhanced Raman scattering signal in plasmonic rod–sphere assemblies.^9,68^

## Conclusion

In this work, we have shown the successful assembly of Au/TiO_2_ sphere–rod mixtures into 3D binary supraparticles with tunable phase behavior. Colloidal membrane supraparticles were formed for a sphere-to-rod number ratio of 5, while lowering this ratio to 0.6 resulted in a binary smectic phase. This is in agreement with numerical simulations for our rod–sphere mixtures showing that the binary smectic phase is stable for sphere-to-rod number ratios up to 1. Above that ratio, the depletion interactions induced by the presence of spheres were not strong enough to continue the 2D single rod layers to self-assemble further into a binary smectic phase, as colloidal membranes were formed instead, stabilized by repulsions between the membranes. Both the membrane and binary smectic phases were observed at the surface as well as in the interior of the supraparticles by using advanced electron microscopy techniques. In addition, the formation of supraparticles was monitored over time by using POM, showing anisotropic phase behavior during the final stages of the self-assembly process. Our binary rod–sphere supraparticles are promising materials for optical, catalytic, and electronic applications, where precise tuning of light absorption and/or charge transport between nanoparticles is essential for maximizing performance.

## Experimental Methods

### TiO_2_ Nanorod Synthesis

Brookite TiO_2_ nanorods were obtained using an adapted version of a previously published seed-mediated procedure.^41–43^ First, 100 mL of oleylamine, 100 mL of 1-octadecene, and 5 mL of oleic acid were added to a 3-neck 500 mL round-bottom flask while stirring at 400 rpm. The reaction mixture was heated at 120 °C for 1 h under vacuum and subsequently cooled down to 60 °C. Once the temperature stabilized, the flask was placed under a N_2_ flow, followed by a swift addition of 15 mL of a stock solution containing 0.2 M TiCl_4_ and 1 M oleic acid in 1-octadene, prepared in a glovebox. Next, the solution was rapidly heated to 290 °C at a ramp of 20 °C/min and held for 30 min to form the anatase seeds. To grow the seeds into brookite TiO_2_ nanorods, 50 mL of stock solution was dropwise injected with a syringe pump at a rate of 0.25 mL/min. After growth, the flask was allowed to cool down to room temperature while stirring. Next, the crude product was centrifugated at 6000 rcf (relative centrifugal force) for 10 min and redispersed in toluene. This purification step was repeated twice using ethanol as an antisolvent to improve the length monodispersity.

The as-synthesized nanorods were post-treated with additional oleic acid and oleylamine ligands to increase the ligand coverage, reducing attractive interparticle interactions.^43^ Briefly, 1 mL of TiO_2_ nanorod dispersion in toluene was added to a 50 mL round-bottom flask containing 5 mL of oleic acid, 5 mL of oleylamine, and 5 mL of 1-octadecene. The flask was heated at 75 °C under vacuum for at least 30 min and left to stir under a N_2_ atmosphere overnight. The nanorods were collected by adding ethanol as an antisolvent, followed by centrifugation at 8000 rcf for 5 min and redispersion in toluene. This step was repeated twice.

### Gold Nanosphere Synthesis

Oleylamine-capped Au nanospheres were obtained following a modified procedure of Peng and Sun et al.,^45^ yielding relatively small nanoparticles around 4 nm. First, 0.5 mmol of HAuCl_4_ · 3 H_2_O was dissolved in 10 mL of oleylamine and 10 mL of octane in a 20 mL glass vial. The solution was transferred to a 50 mL round-bottom flask and heated at 30 °C under a N_2_ atmosphere. The solution was left stirring at 400 rpm for 30 min. Next, the stirring was increased to 1200 rpm, followed by a rapid injection of a TBAB solution (87 mg of TBAB in 1 mL of oleylamine and 1 mL of octane), which immediately turned the mixture black. The mixture was left for 2 h, ultimately turning the color to deep red, indicating the formation of small Au nanoparticles. Subsequently, antisolvent acetone was slowly added to the crude product, followed by centrifugation at 5000 rcf for 5 min. The supernatant was discarded and the isolated precipitate was redispersed in toluene. This step was repeated once with ethanol as an antisolvent. The resulting Au nanospheres were stored in dark until further use.

### Formation of Au/TiO_2_ Binary Superlattices

The self-assembly of TiO_2_ nanorods and Au nanospheres into binary superlattices was carried out using a self-assembly procedure for the assembly of binary NPs dispersed in an apolar liquid on the surface of a polar liquid for the creation of 2D or thin 3D colloidal crystals (specifically, it was demonstrated for FePt/Fe_3_O_4_ superlattices, but has been applied for many different NP systems subsequently).^48^ Generally, TiO_2_ nanorods and Au nanospheres were added together into a 1 mL glass vial and further diluted with toluene until a final volume of 50 μL was reached. The nanoparticles dispersion was vortex mixed (IKA Vortex 3) and dropwise added on top of 2 mL of water in a 3 cm glass Petri dish. The Petri dish was then covered with a bigger Petri dish and left for several hours. Once the nanoparticles were fully dried on the water–air interface, the resulting thin film was transferred to a Formvar/carbon-coated copper grid (200 mesh, Ted Pella) for structural analysis.

### Formation of Au/TiO_2_ Supraparticles

The Au/TiO_2_ supraparticles were obtained by evaporating oil-in-water (O/W) emulsion droplets, causing close packing of nanoparticles.^38,69^ Typically, TiO_2_ nanorods and Au nanospheres, both dispersed in toluene, were mixed together in a 1 mL glass vial and diluted with toluene to obtain a final volume of 150 μL. The mixture was mixed with a vortex mixer and added to 10 mL of 8 mM SDS solution in a 20 mL glass vial presaturated with 20 μL of toluene. The mixture was emulsified using a Turrax mixer (IKA T25 digital, Ultra Turrax) at 3000 rpm for 20 s. The glass vial was quickly covered with Teflon tape. A needle was used to make 1 hole to allow toluene to evaporate. The vial was left on an orbital shaker (IKA KS260 basic) for 3 days, shaking at 250 rpm, turning the pink turbid emulsion to a pink transparent dispersion of supraparticles. The supraparticles were collected by sequential centrifugation at 50 rcf and 250 rcf, each for 5 min to prevent larger supraparticles from breaking due to the centrifugal force. The precipitate was isolated and redispersed in water. This purification step was repeated twice.

### Ultramicrotomy

Ultramicrotomy sections were prepared by sectioning resin-embedded supraparticles using a Reichert/Leica UltraCut E ultramicrotome. Dried supraparticles were previously embedded in EpoFix resin and left to harden at 60 °C overnight. Next, the resin was modified with a razor blade to expose the sample. The resin was mounted on the ultramicrotome equipped with a DiATOME diamond knife. Initial 80 nm sections were cut to ensure uniform thickness, followed by 50–70 nm thin sections prepared at a cutting speed of 1 mm/s. These were collected onto Formvar/carbon-coated copper TEM grids for structural analysis.

### Simulations

The simulations were performed using the Large-scale Atomic/Molecular Massively Parallel Simulator (LAMMPS 21Nov2023)^70^ in the NPT ensemble. Particle interactions are described by the purely repulsive Weeks–Chandler–Andersen (WCA) potential with interaction strength ε = 40k_B_T. The spheres have a diameter σ, and the rods are represented as rigid bodies composed of ten nonoverlapping spheres of diameter σ, resulting in an aspect ratio of *L*/σ = 10. The initial configurations are created by placing 17,500 rods on a regular 3D grid. Subsequently, rods are randomly replaced by spheres to obtain the desired sphere-to-rod number ratio while keeping the total number of particles constant, see Figure S6. The imposed pressure controls the packing fraction. After equilibration, all relevant quantities are averaged over 10 configurations, sampled every 10^5^ timesteps.

To further quantify the local nematic order, the structure of the mixture is characterized using local nematic and smectic order parameters.^26^ The local nematic order parameter *S*_*i*_ of particle *i* is defined as

$$S_{i}=\frac{1}{n_{i}}\sum_{j=1}^{n_{i}}\left[\frac{3}{2}{\left(u_{i}\cdot u_{j}\right)}^{2}-\frac{1}{2}\right]$$

where *n*_*i*_ is the number of neighbors of particle *i*. Two particles are considered neighbors if their surface-to-surface distance satisfies ρ_*ij*_ < 1.5 σ. By definition, *S*_*i*_ ranges from −0.5 (rods oriented perpendicular to its neighbors) to 1 (perfect alignment). The local smectic order parameter τ_*i*_ is defined as

$$\tau_{i}=S_{i}\left[1-\left(\frac{1}{m_{i}}\sum_{j=1}^{m_{i}}\left|\frac{r_{ij}}{r_{cut}}\cdot u_{i}\right|\right)\right]$$

where **r**_*ij*_ is the vector connecting the centers of mass of rod *i* and *j* pair, *r*_cut_ = *L*/2 denotes the cutoff distance, and *m*_*i*_ is the number of neighbors of particle *i* that satisfy |**r**_*ij*_|<*r*_cut_. By construction, τ_*i*_ ranges from −0.5 (particle transverse and not in line with its neighbors) to 1 (perfect alignment and in line with neighbors).

## Supplementary Material















## Notes

Experimental details including chemicals and physical characterization; particle size determination; particle ratio calculations; and supplementary figures (PDF)
Tomography alignment of an ultramicrotomy section of a binary smectic supraparticle (MP4)
Simulation of binary mixtures of rods and spheres for different packing fractions (MP4)
Simulation of the nucleation of a binary smectic phase (MP4)
Simulation of spheres and rods unmixed in the rod axial direction (MP4)
Simulation of spheres and rods unmixed in the direction transversal to the rod axis (MP4)
Supplementary 3D reconstruction of a binary smectic supraparticle HTML file (ZIP)

## Abbreviations

BF-TEM: bright-field transmission electron microscopy
BSE: backscattered electrons
EDX: energy dispersive X-ray
POM: polarized optical microscopy
SE: secondary electrons
SEM: scanning electron microscopy
HAADF-STEM: high-angle annular dark-field scanning transmission electron microscopy.
σ: sphere diameter and length scale;
*V*_rod_ (*V*_sph_): rod (sphere) volume;
*N*: total number of particles;
*N*_rod_ (*N*_sph_): number of rods (spheres);
*x*_sph_: fraction of spheres;
*I*: light intensity;
λ: wavelength;
θ: angle between the polarizer and the optical axis;
Δ*n*: refractive index difference;
*Q*: nematic order tensor;
n: nematic director;
*S*: nematic order parameter;
τ: smectic order parameter;
τ_*i*_: local smectic order parameter of rod *i*;
*d*: smectic layer spacing and sample thickness;
u_*i*_: unit vector describing the orientation of the long axis of rod *i*;
δ_αβ_: Kronecker delta.

## References

[1] Wintzheimer S., Reichstein J., Groppe P., Wolf A., Fett B., Zhou H., Pujales-Paradela R., Miller F., Müssig S., Wenderoth S., Mandel K. (2021). Supraparticles for Sustainability. Adv. Funct. Mater..

[2] Wang Y., Wise A. K., Tan J., Maina J. W., Shepherd R. K., Caruso F. (2014). Mesoporous Silica Supraparticles
for Sustained Inner-Ear
Drug Delivery. Small.

[3] Nakayama Y., Kämäräinen T., Uchiyama H., Tozuka Y., Kadota K. (2025). Role of Amyloid Nanofibrils
in Drug Incorporation and
Release from Wrinkled Supraparticles. ACS Appl.
Nano Mater..

[4] Yu Y., Yang X., Liu M., Nishikawa M., Tei T., Miyako E. (2019). Amphipathic Nanodiamond Supraparticles for Anticancer
Drug Loading and Delivery. ACS Appl. Mater.
Interfaces.

[5] Hou K., Han J., Tang Z. (2020). Formation of Supraparticles and Their Application in
Catalysis. ACS Mater. Lett..

[6] Ro G., Hwang D. K., Kim Y. (2019). Hydrogen Generation
Using Pt/Ni Bimetallic
Nanoparticles Supported on Fe3O4@SiO2@TiO2Multi-Shell Microspheres. J. Ind. Eng. Chem..

[7] Luneau M., Shirman T., Filie A., Timoshenko J., Chen W., Trimpalis A., Flytzani-Stephanopoulos M., Kaxiras E., Frenkel A. I., Aizenberg J., Friend C. M., Madix R. J. (2019). Dilute Pd/Au Alloy
Nanoparticles
Embedded in Colloid-Templated Porous SiO2: Stable Au-Based Oxidation
Catalysts. Chem. Mater..

[8] Su L., Han D., Zhu G., Xu H., Luo W., Wang L., Jiang W., Dong A., Yang J. (2019). Tailoring the Assembly
of Iron Nanoparticles in Carbon Microspheres toward High-Performance
Electrocatalytic Denitrification. Nano Lett..

[9] van
der Hoeven J. E. S., Gurunarayanan H., Bransen M., de Winter D. A. M., de Jongh P. E., van Blaaderen A. (2022). Silica-Coated Gold Nanorod Supraparticles:
A Tunable Platform for Surface Enhanced Raman Spectroscopy. Adv. Funct. Mater..

[10] Shen Y., Wang W., Chen X., Zhang B., Wei D., Gao S., Cui B. (2016). Nitrogen Dioxide Sensing Using Tungsten Oxide Microspheres
with Hierarchical Nanorod-Assembled Architectures by a Complexing
Surfactant-Mediated Hydrothermal Route. J. Mater.
Chem. A.

[11] Suematsu K., Shin Y., Hua Z., Yoshida K., Yuasa M., Kida T., Shimanoe K. (2014). Nanoparticle
Cluster Gas Sensor:
Controlled Clustering of SnO2 Nanoparticles for Highly Sensitive Toluene
Detection. ACS Appl. Mater. Interfaces.

[12] Mistlberger G., Medina-Castillo A. L., Borisov S. M., Mayr T., Fernández-Gutiérrez A., Fernandez-Sanchez J. F., Klimant I. (2011). Mini-Emulsion Solvent Evaporation:
A Simple and Versatile Way to Magnetic Nanosensors. Microchim. Acta.

[13] Cao Z., Chen X., Xing L., Liao Y., Xu M., Li X., Liu X., Li W. (2018). Nano-MnO2@TiO2 Microspheres: A Novel
Structure and Excellent Performance as Anode of Lithium-Ion Batteries. J. Power Sources.

[14] Wang J., Sultan U., Goerlitzer E. S. A., Mbah C. F., Engel M., Vogel N. (2020). Structural Color of
Colloidal Clusters as a Tool to Investigate Structure
and Dynamics. Adv. Funct. Mater..

[15] Bao L., You H., Wang L., Li L., Qiao R., Zhang Y., Zhong Y., Xiong Y., Li Z. (2014). Self-Assembly
of LaF3:Yb,Er/Tm
Nanoplates into Colloidal Spheres and Tailoring Their Upconversion
Emissions with Fluorescent Dyes. J. Mater. Chem.
C.

[16] Shevchenko E. V., Talapin D. V., Murray C. B., O’Brien S. (2006). Structural
Characterization of Self-Assembled Multifunctional Binary Nanoparticle
Superlattices. J. Am. Chem. Soc..

[17] Urban J. J., Talapin D. V., Shevchenko E. V., Kagan C. R., Murray C. B. (2007). Synergism
in Binary Nanocrystal Superlattices Leads to Enhanced P-Type Conductivity
in Self-Assembled PbTe/Ag2Te Thin Films. Nat.
Mater..

[18] Zhou W., Li Y., Partridge B. E., Mirkin C. A. (2024). Engineering Anisotropy into Organized
Nanoscale Matter. Chem. Rev..

[19] Deng K., Luo Z., Tan L., Quan Z. (2020). Self-Assembly of Anisotropic Nanoparticles
into Functional Superstructures. Chem. Soc.
Rev..

[20] Onsager L. (1949). The Effects
of Shape on the Interaction of Colloidal Particles. Ann. N.Y. Acad. Sci..

[21] Lekkerkerker H. N. W., Vroege G. J. (2013). Liquid Crystal Phase
Transitions in Suspensions of
Mineral Colloids: New Life from Old Roots. Philos.
Trans. R. Soc. Math. Phys. Eng. Sci..

[22] Koda T., Numajiri M., Ikeda S. (1996). Smectic-A Phase of a Bidisperse System
of Parallel Hard Rods and Hard Spheres. J. Phys.
Soc. Jpn..

[23] Adams M., Dogic Z., Keller S. L., Fraden S. (1998). Entropically Driven
Microphase Transitions in Mixtures of Colloidal Rods and Spheres. Nature.

[24] Ye X., Millan J. A., Engel M., Chen J., Diroll B. T., Glotzer S. C., Murray C. B. (2013). Shape Alloys of Nanorods and Nanospheres
from Self-Assembly. Nano Lett..

[25] Dogic Z., Frenkel D., Fraden S. (2000). Enhanced Stability
of Layered Phases
in Parallel Hard Spherocylinders Due to Addition of Hard Spheres. Phys. Rev. E: Stat., Nonlinear, Soft Matter Phys..

[26] Bakker H. E., Dussi S., Droste B. L., Besseling T. H., Kennedy C. L., Wiegant E. I., Liu B., Imhof A., Dijkstra M., van Blaaderen A. (2016). Phase Diagram
of Binary Colloidal
Rod-Sphere Mixtures from a 3D Real-Space Analysis of Sedimentation–Diffusion
Equilibria. Soft Matter.

[27] Cuetos A., Sanz E., Dijkstra M. (2010). Can the Isotropic-Smectic Transition
of Colloidal Hard Rods Occur via Nucleation and Growth?. Faraday Discuss..

[28] Ni R., Belli S., van Roij R., Dijkstra M. (2010). Glassy Dynamics, Spinodal
Fluctuations, and the Kinetic Limit of Nucleation in Suspensions of
Colloidal Hard Rods. Phys. Rev. Lett..

[29] Dogic Z., Fraden S. (2001). Development of Model
Colloidal Liquid Crystals and
the Kinetics of the Isotropic–Smectic Transition. Philos. Trans. R. Soc. Math. Phys. Eng. Sci..

[30] Kuijk A., Byelov D. V., Petukhov A. V., van Blaaderen A., Imhof A. (2012). Phase Behavior of Colloidal Silica
Rods. Faraday
Discuss..

[31] Barry E., Dogic Z. (2010). Entropy Driven Self-Assembly
of Nonamphiphilic Colloidal Membranes. Proc.
Natl. Acad. Sci. U S A.

[32] Kang L., Gibaud T., Dogic Z., Lubensky T. C. (2016). Entropic Forces
Stabilize Diverse Emergent Structures in Colloidal Membranes. Soft Matter.

[33] Yang Y., Barry E., Dogic Z., Hagan M. F. (2012). Self-Assembly of
2D Membranes from Mixtures of Hard Rods and Depleting Polymers. Soft Matter.

[34] Ge J., Hu Y., Biasini M., Beyermann W. P., Yin Y. (2007). Superparamagnetic Magnetite
Colloidal Nanocrystal Clusters. Angew. Chem.,
Int. Ed..

[35] Montanarella F., Urbonas D., Chadwick L., Moerman P. G., Baesjou P. J., Mahrt R. F., van Blaaderen A., Stöferle T., Vanmaekelbergh D. (2018). Lasing Supraparticles Self-Assembled from Nanocrystals. ACS Nano.

[36] Choi Y. H., Kim S.-H. (2023). Production of Photonic Supraballs Composed of Single-Crystalline
Colloidal Arrays through Osmosis-Induced Consolidation. Chem. Mater..

[37] Vanmaekelbergh D., van Vugt L. K., Bakker H. E., Rabouw F. T., de Nijs B., van Dijk-Moes R. J. A., van Huis M. A., Baesjou P. J., van Blaaderen A. (2015). Shape-Dependent
Multiexciton Emission and Whispering Gallery Modes in Supraparticles
of CdSe/Multishell Quantum Dots. ACS Nano.

[38] de
Nijs B., Dussi S., Smallenburg F., Meeldijk J. D., Groenendijk D. J., Filion L., Imhof A., van Blaaderen A., Dijkstra M. (2015). Entropy-Driven Formation of Large Icosahedral Colloidal
Clusters by Spherical Confinement. Nat. Mater..

[39] Panayotov D. A., Frenkel A. I., Morris J. R. (2017). Catalysis
and Photocatalysis by Nanoscale
Au/TiO2: Perspectives for Renewable Energy. ACS Energy Lett..

[40] Miller D. S., Carlton R. J., Mushenheim P. C., Abbott N. L. (2013). Instructional Review:
An Introduction to Optical Methods for Characterizing Liquid Crystals
at Interfaces. Langmuir ACS J. Surf. Colloids.

[41] Buonsanti R., Grillo V., Carlino E., Giannini C., Kipp T., Cingolani R., Cozzoli P. D. (2008). Nonhydrolytic Synthesis of High-Quality
Anisotropically Shaped Brookite TiO2 Nanocrystals. J. Am. Chem. Soc..

[42] Cargnello M., Montini T., Smolin S. Y., Priebe J. B., Delgado
Jaén J. J., Doan-Nguyen V. V. T., McKay I. S., Schwalbe J. A., Pohl M.-M., Gordon T. R., Lu Y., Baxter J. B., Brückner A., Fornasiero P., Murray C. B. (2016). Engineering Titania
Nanostructure to Tune and Improve Its Photocatalytic Activity. Proc. Natl. Acad. Sci. U S A.

[43] Hosseini S. N., Grau-Carbonell A., Nikolaenkova A. G., Xie X., Chen X., Imhof A., van Blaaderen A., Baesjou P. J. (2020). Smectic Liquid Crystalline
Titanium Dioxide Nanorods: Reducing Attractions by Optimizing Ligand
Density. Adv. Funct. Mater..

[44] Arenas
Esteban D., Wang D., Kadu A., Olluyn N., Sánchez-Iglesias A., Gomez-Perez A., González-Casablanca J., Nicolopoulos S., Liz-Marzán L. M., Bals S. (2024). Quantitative 3D Structural Analysis
of Small Colloidal Assemblies under Native Conditions by Liquid-Cell
Fast Electron Tomography. Nat. Commun..

[45] Peng S., Lee Y., Wang C., Yin H., Dai S., Sun S. (2008). A Facile Synthesis
of Monodisperse Au Nanoparticles and Their Catalysis of CO Oxidation. Nano Res..

[46] Bransen, M. Measuring interactions between colloidal (nano)­particles, PhD thesis. 2024, DOI: 10.33540/2252.

[47] Diroll B. T., Weigandt K. M., Jishkariani D., Cargnello M., Murphy R. J., Hough L. A., Murray C. B., Donnio B. (2015). Quantifying
“Softness” of Organic Coatings on Gold Nanoparticles
Using Correlated Small-Angle X-Ray and Neutron Scattering. Nano Lett..

[48] Dong A., Chen J., Vora P. M., Kikkawa J. M., Murray C. B. (2010). Binary
Nanocrystal Superlattice Membranes Self-Assembled at the Liquid–Air
Interface. Nature.

[49] Sultan U., Götz A., Salcedo N., Sandner L., Beunen J. M. P., Kichigin A., Vorndran C., Malgaretti P., Apeleo Zubiri B., Thommes M., Harting J., Spiecker E., Vogel N. (2025). Open the Pores: Particles with Fully Accessible Hierarchical Pore
Networks by Controlling Phase Separation in Confinement. J. Am. Chem. Soc..

[50] Li M., Deng Y., Wu G., Xue S., Yan Y., Liu Z., Zou J., Yang D., Dong A. (2021). Multi-Chambered, Carbon-Coated
Ni0.4Fe2.6O4 Nanoparticle Superlattice Microspheres for Boosting Water
Oxidation Reaction. Aggregate.

[51] Chen C., Palacio-Betancur V., Norouzi S., Zubieta-Rico P. F., Chang N., Sadati M., Rowan S. J., de Pablo J. J. (2024). LCPOM:
Precise Reconstruction of Polarized Optical Microscopy Images of Liquid
Crystals. Chem. Mater..

[52] Bhupathi P., Jaworski L., Hwang J., Tanner D. B., Obukov S., Lee Y., Mulders N. (2010). Optical Birefringence
in Uniaxially Compressed Aerogels. New J. Phys..

[53] Arakawa Y., Nakajima S., Ishige R., Uchimura M., Kang S., Konishi G., Watanabe J. (2012). Synthesis
of Diphenyl-Diacetylene-Based
Nematic Liquid Crystals and Their High Birefringence Properties. J. Mater. Chem..

[54] Chowdhury R. A., Peng S. X., Youngblood J. (2017). Improved Order Parameter (Alignment)
Determination in Cellulose Nanocrystal (CNC) Films by a Simple Optical
Birefringence Method. Cellulose.

[55] Martınez-Ratón Y., Cinacchi G., Velasco E., Mederos L. (2006). Depletion Effects in
Smectic Phases of Hard-Rod-Hard-Sphere Mixtures. Eur. Phys. J. E.

[56] Lei D., Wang H., Zheng Z., Wu F., Wan S., Yang D., Li W., Li T., Dong A. (2026). Pathway-Engineered
Co-Assembly of Nanorod–Nanosphere Binary Superlattices. Chem. Commun..

[57] Ma L., Liu H., Mei B., Chen J., Cheng Q., Ma J., Yang B., Li Q., Yang H. (2025). Cu Supraparticles with
Enhanced Mass Transfer and Abundant C-C Coupling Sites Achieving Ampere-Level
CO2-to-C2+ Electrosynthesis. Nat. Commun..

[58] Plunkett A., Eldridge C., Schneider G. A., Domènech B. (2020). Controlling
the Large-Scale Fabrication of Supraparticles. J. Phys. Chem. B.

[59] Wu J., Yadavali S., Lee D., Issadore D. A. (2021). Scaling up the Throughput
of Microfluidic Droplet-Based Materials Synthesis: A Review of Recent
Progress and Outlook. Appl. Phys. Rev..

[60] Naveenkumar P. M., Roemling L. J., Sultan U., Vogel N. (2024). Fabrication of Spherical
Colloidal Supraparticles via Membrane Emulsification. Langmuir.

[61] Wang T., Zhuang J., Lynch J., Chen O., Wang Z., Wang X., LaMontagne D., Wu H., Wang Z., Cao Y. C. (2012). Self-Assembled Colloidal Superparticles
from Nanorods. Science.

[62] Nagaoka Y., Wang T., Lynch J., LaMontagne D., Cao Y. C. (2012). Binary Assembly of Colloidal Semiconductor Nanorods
with Spherical Metal Nanoparticles. Small.

[63] Sánchez-Iglesias A., Grzelczak M., Pérez-Juste J., Liz-Marzán L.
M. (2010). Binary
Self-Assembly of Gold Nanowires with Nanospheres and Nanorods. Angew. Chem., Int. Ed..

[64] Chen C., Nan C., Wang D., Su Q., Duan H., Liu X., Zhang L., Chu D., Song W., Peng Q., Li Y. (2011). Mesoporous Multicomponent Nanocomposite Colloidal Spheres: Ideal
High-Temperature Stable Model Catalysts. Angew.
Chem., Int. Ed..

[65] Leschkies K. S., Divakar R., Basu J., Enache-Pommer E., Boercker J. E., Carter C. B., Kortshagen U. R., Norris D. J., Aydil E. S. (2007). Photosensitization of ZnO Nanowires
with CdSe Quantum Dots for Photovoltaic Devices. Nano Lett..

[66] Xue B., Li T., Wang B., Ji L., Yang D., Dong A. (2018). Self-Assembled
Fe 3 O 4 Nanoparticle-Doped TiO 2 Nanorod Superparticles with Highly
Enhanced Lithium Storage Properties. Sustain.
Energy Fuels.

[67] Zhang Y., Xiang Q., Xu J., Xu P., Pan Q., Li F. (2009). Self-Assemblies of Pd Nanoparticles
on the Surfaces of Single Crystal
ZnO Nanowires for Chemical Sensors with Enhanced Performances. J. Mater. Chem..

[68] Lee S. J., Baik J. M., Moskovits M. (2008). Polarization-Dependent Surface-Enhanced
Raman Scattering from a Silver-Nanoparticle-Decorated Single Silver
Nanowire. Nano Lett..

[69] Wang D., Dasgupta T., van der Wee E. B., Zanaga D., Altantzis T., Wu Y., Coli G. M., Murray C. B., Bals S., Dijkstra M., van Blaaderen A. (2021). Binary Icosahedral Clusters of Hard Spheres in Spherical
Confinement. Nat. Phys..

[70] Thompson A. P., Aktulga H. M., Berger R., Bolintineanu D. S., Brown W. M., Crozier P. S., in ’t
Veld P. J., Kohlmeyer A., Moore S. G., Nguyen T. D., Shan R., Stevens M. J., Tranchida J., Trott C., Plimpton S. J. (2022). LAMMPS
- a Flexible Simulation Tool for Particle-Based Materials Modeling
at the Atomic, Meso, and Continuum Scales. Comput.
Phys. Commun..

