# Supplementary material for: Self-Assembly of Au/TiO2 Sphere–Rod Mixtures into Binary Supraparticles
Source: ACS Nano. 2026 Sep 25;20(40):27415–27. doi: 10.1021/acsnano.6c12966 (PMC13671251; doi:10.1021/acsnano.6c12966)
Supplement: Supplementary file 1 [file nn6c12966_si_001.pdf]

Self-assembly of Au/TiO_2 sphere-rod mixtures into binary supraparticles

Marianne Bijl[a,b,], André F. V. Matias[b], Lukáš Kolácný[c], Erik Betz-Güttner[a], Martin Veselý[c], Marjolein Dijkstra[b], Jessi E. S. van der Hoeven[a,*] and Alfons van Blaaderen[b,*]*

[a] Materials Chemistry and Catalysis, Debye Institute for Nanomaterials Science, Utrecht University, Universiteitsweg 99, 3584 CG, Utrecht, The Netherlands

[b] Soft Condensed Matter & Biophysics Group, Debye Institute for Nanomaterials Science, Utrecht University, Princetonplein 1, 3584 CC, Utrecht, The Netherlands

[c] Department of Organic Technology, University of Chemistry and Technology Prague, Technická 5, 166 28, Prague, Czech Republic

[*]Corresponding authors

E-mail: m.bijl1@uu.nl, j.e.s.vanderhoeven@uu.nl, a.vanblaaderen@uu.nl

1. Experimental details

1.1 Chemicals

All chemicals were used as received without further purification. Acetone, gold (III) chloride trihydrate ($HAuCl_4 \cdot 3H_2O$, ≥99.9%), octane, oleic acid (90%), oleylamine (80-90% C_{18} content), sodium dodecyl sulphate (SDS), tert-butylamine borane (TBAB, 97%), titanium(IV) chloride ($TiCl_4$, 99%) and toluene were purchased from Sigma Aldrich. 1-octadecene (90%) was obtained from Thermo Scientific Acros, and absolute ethanol was obtained from Fisher Scientific. Ultrapure deionised water (Millipore Milli-Q grade, 18.2 MΩ cm^{-1}) was used and all glassware was cleaned with fresh aqua regia (HCl/HNO_3, 3 : 1 volume ratio) before use.

1.2 Physical characterization

The samples were characterized with scanning electron microscopy (SEM), bright-field transmission electron microscopy (BF-TEM), scanning transmission electron microscopy (STEM) tomography and energy-Dispersive X-ray spectroscopy (EDX), polarized optical light microscopy (POM), and inductively coupled plasma-mass spectrometry (ICP-MS). Details are presented below.

Scanning electron microscopy. SEM images were obtained with a secondary electron detector and backscattered electron detector using a Helios G3UC operating at an acceleration voltage of 15 kV and working distance of 4 nm. Prior to SEM imaging, samples were dried on a Si wafer, immobilized on the sample stub with carbon tape.

Transmission electron microscopy. BF-TEM images were acquired with a Talos L120C microscope operating with an acceleration voltage of 120 kV. Prior to TEM imaging, samples drop-casted onto a Formvar/carbon-coated copper grid (200 mesh, Ted Pella). The EDX and tomography measurements were performed in STEM-HAADF (scanning transmission electron microscopy-high angle annular dark field) imaging mode on a Talos F200X, operated at 200 kV. The elemental maps were acquired at a camera length of 260 mm with a dwell time of 2 µs over a 2048 x 2048 pixel area. A total acquisition time of 10 minutes was used for each elemental map. For the tomography measurements, a series of images were collected with 2° increments. Each tilt image was recorded at a resolution of 2048 × 2048 pixels with a dwell time of 2 µs and a total frame time of 11.79 s. The tomography data were reconstructed with IMOD software using an iterative algebraic reconstruction technique.[1] The Au nanoparticles present in the sample were used as fiducial markers. A top-hat transformation was used for segmentation of the TiO_2 nanorods and histogram thresholding was used for the Au nanoparticles in Avizo 3D.

Polarized optical microscopy. Emulsion droplets, prepared as described in the Experimental methods, were transferred to a 3 cm diameter glass Petri dish and covered with glass coverslips. The cross-polarized optical microscopy images were obtained with a Nikon Eclipse Ti-E inverted microscope equipped with a Nikon DS-Ri2 CMOS camera, a T-P2 DIC Polarizer module and an analysis polarizer. Corresponding bright-field optical images were obtained by removing the polarizing filters. The emulsion droplets were initially imaged at the water–air (t = 0-15 min), while the subsequent evaporation into supraparticles was captured at the bottom of the Petri dish

due to sedimentation (t = 16-90 min). For analysis of the light intensity, the microscopy RGB images were converted to 8-bit grayscale using standard luminance weighting in ImageJ (version 1.53e).[2] This conversion combined the three colour channels (red, green and blue) into a single intensity value per pixel, which was subsequently averaged over all pixel values of the image, providing a mean grey value that represents the overall brightness.

2. Supporting data

2.1 Particle size determination

The average size of the individual Au nanospheres and TiO_2 nanorods were determined on BF-TEM images, obtained on a Talos L120C microscope. The size of supraparticles was determined with SEM images, obtained on a Helios G3UC microscope. The polydispersity is based on the relative width of the size distribution.

2.2 Particle ratio (N_{Au}/N_{TiO_2}) calculations

The particle ratio (N_{Au}/N_{TiO_2}) was calculated based on nanoparticle size, density, and concentration.

The Au nanospheres had an average core diameter of 3.5 nm (Figure 1). This corresponds to a volume of 2.4 x 10^{-20} cm^3, calculated using the formula for the volume of a sphere ($V = (4/3)\pi r^3$), and a core mass of 4.3 x 10^{-19} g, based on the density of gold (19.3 g/cm^3). Using the interparticle distance of 1.9 nm, the mass of the ligands (using the density of oleylamine, 0.81 g/cm^3) was 4.9 x 10^{-20} cm^3, giving a total mass of 4.8 x 10^{-19} g. By drying the synthesized Au samples, the concentration in mg/mL was determined, which was used to calculate the amount of particles/mL sample.

The TiO_2 nanorods had average core dimensions of 49 nm (length) and 3.5 nm (diameter), determined from TEM images (Figure 1). The core volume was calculated using the cylindrical volume formula ($V = \pi r^2 h$) to be 4.8 x 10^{-19} cm^3. With the density of brookite TiO_2 (4.2 g/cm^3), the core mass was 2.0 x 10^{-18} g. Including the surface ligands, the nanorod dimensions increased to 53 nm × 6.2 nm, giving a total volume of 1.6 x 10^{-18} cm^3. The ligand shell volume was thus 1.1 x 10^{-18} cm^3. Assuming a 1:1 mixture of oleic acid (0.89 g/cm^3) and oleylamine (0.81 g/cm^3), the ligand mass was calculated to be 9.5 x 10^{-19} g. The total mass of a single ligand-coated TiO_2 nanorod was therefore 3.0 x 10^{-18} cm^3. Using the concentration of the TiO_2 sample, the amount of particles/mL sample was determined.

The particle ratio was ultimately determined by dividing the amount of Au particles over the amount of TiO_2 particles for each supraparticle batch. The theoretical Au weight loading was calculated by dividing the effective mass of Au over the total effective mass of Au and TiO_2.

2.3 Supplementary figures

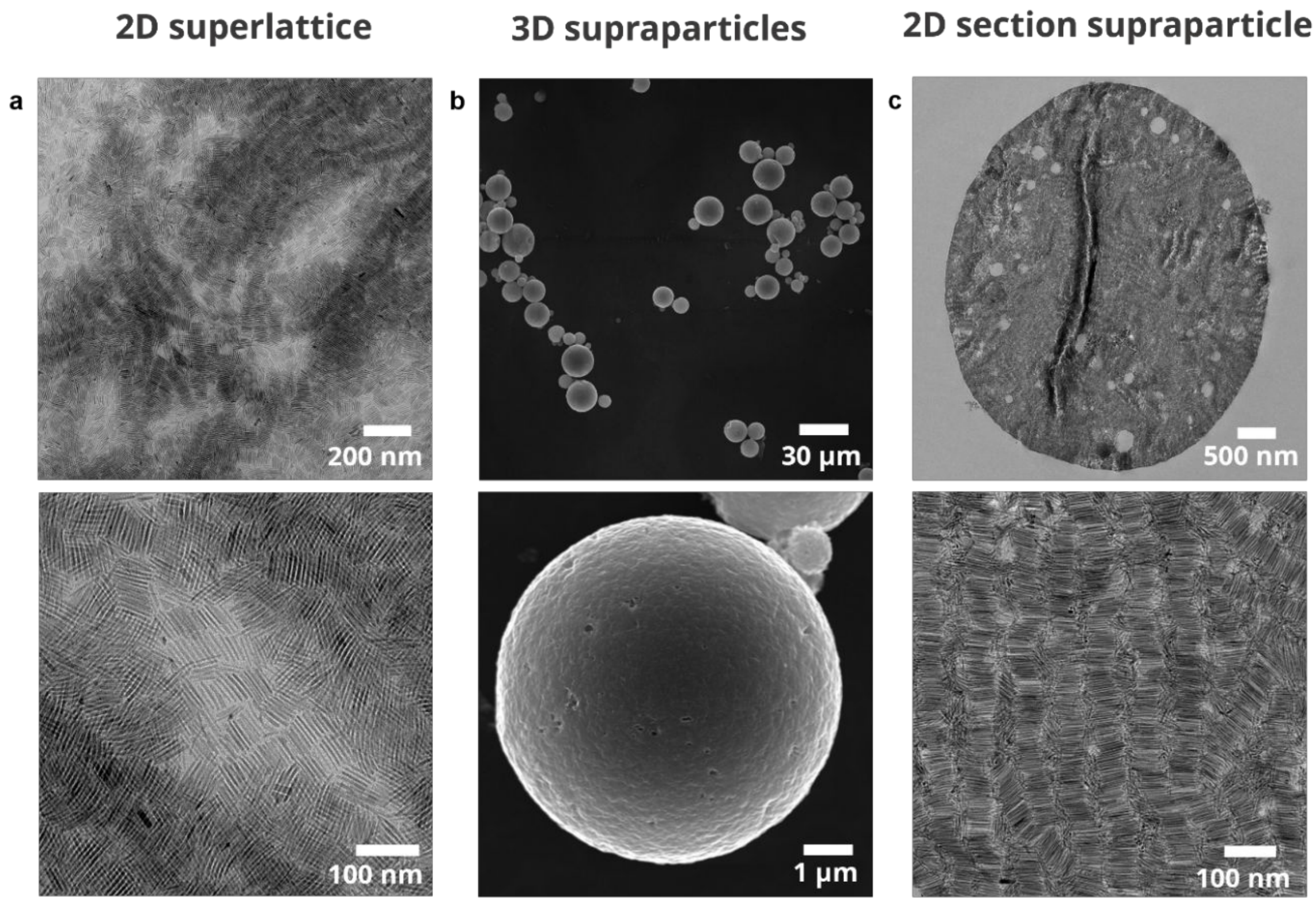

Figure S1: 2D and 3D assembly of TiO_2 nanorods. a) BF-TEM images of TiO_2 superlattices at low and high magnification. b) SEM images acquired with secondary electrons of TiO_2 supraparticles at low and high magnification. c) BF-TEM images of an ultramicrotomy section of a TiO_2 supraparticle at low and high magnification.

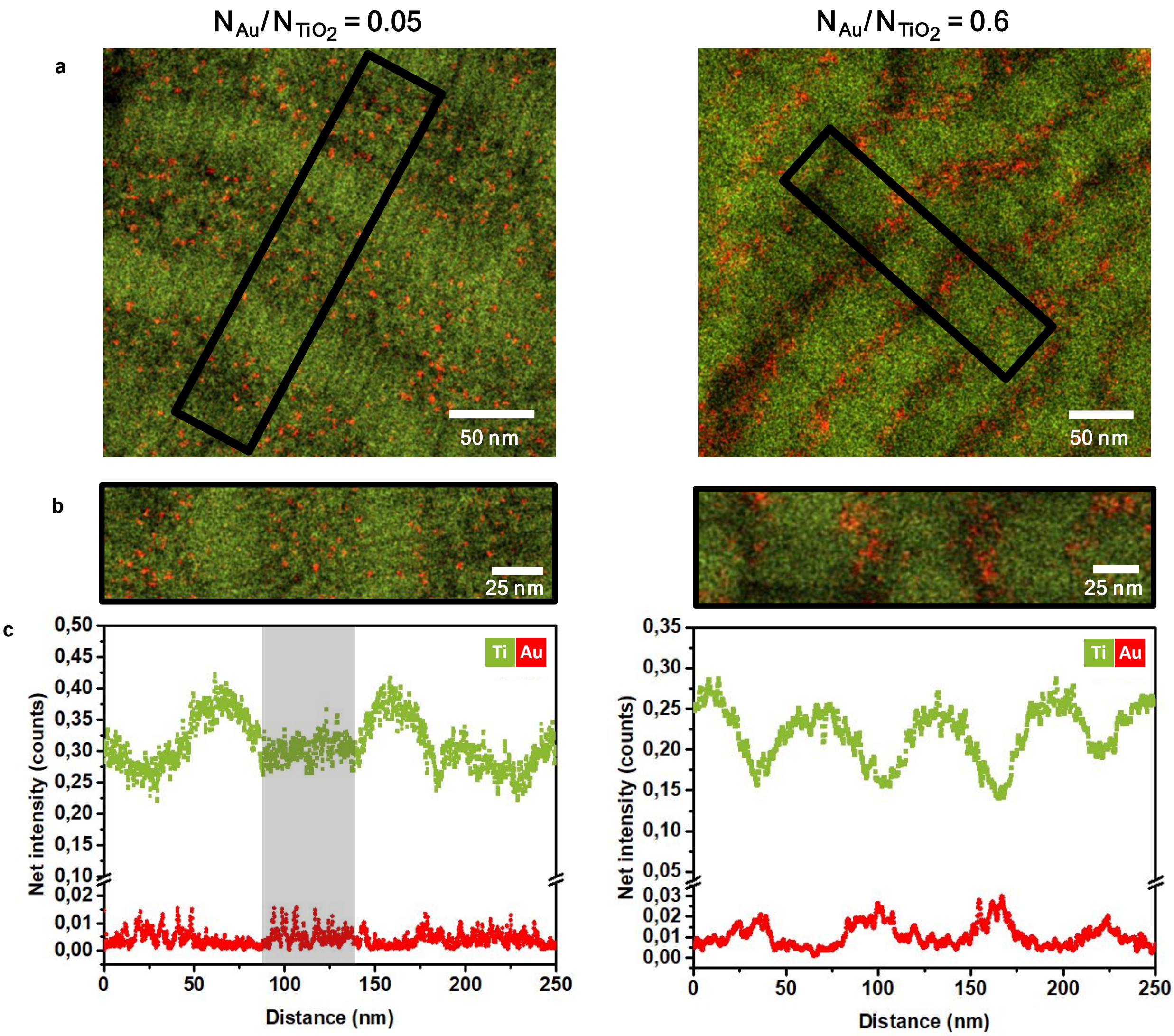

Figure S2: The nanoparticle distribution of Au and TiO_2 nanoparticles determined with EDX with Ti in green and Au in red. a, b) EDX elemental map obtained on a ultramicrotomy section of a Au/TiO_2 supraparticle with particle number ratios (N_{Au}/N_{TiO_2}) of 0.05 (left) and 0.6 (right). c) Corresponding EDX line scans in which the smectic gap for N_{Au}/N_{TiO_2} of 0.05 is highlighted.

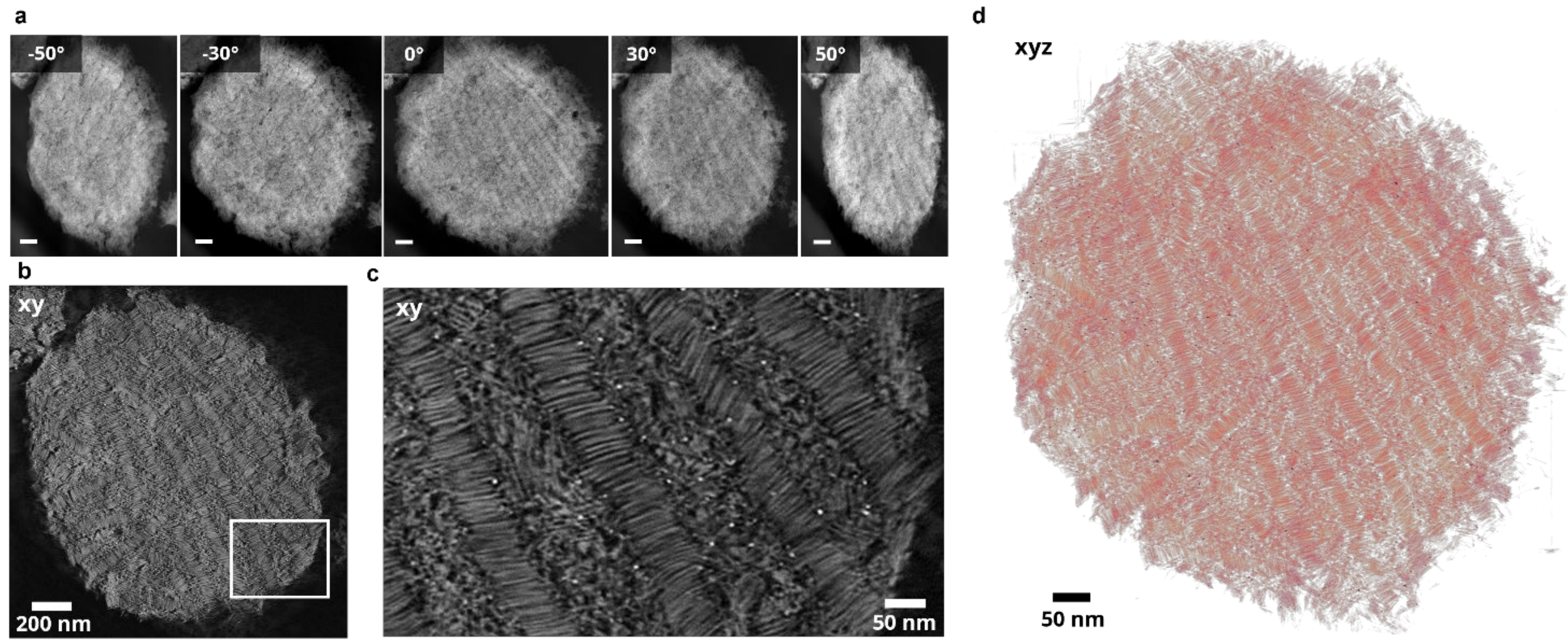

Figure S3: 3D analysis of a Au/TiO_2 supraparticle with $N_{Au}/N_{TiO_2} = 0.05$ using HAADF-STEM tomography. a) A ultramicrotomy section of the supraparticle from -50° to +50° with scale bars of 100 nm. b, c) A reconstructed 2D slice at low and high magnification and d) corresponding 3D reconstruction of the supraparticle.

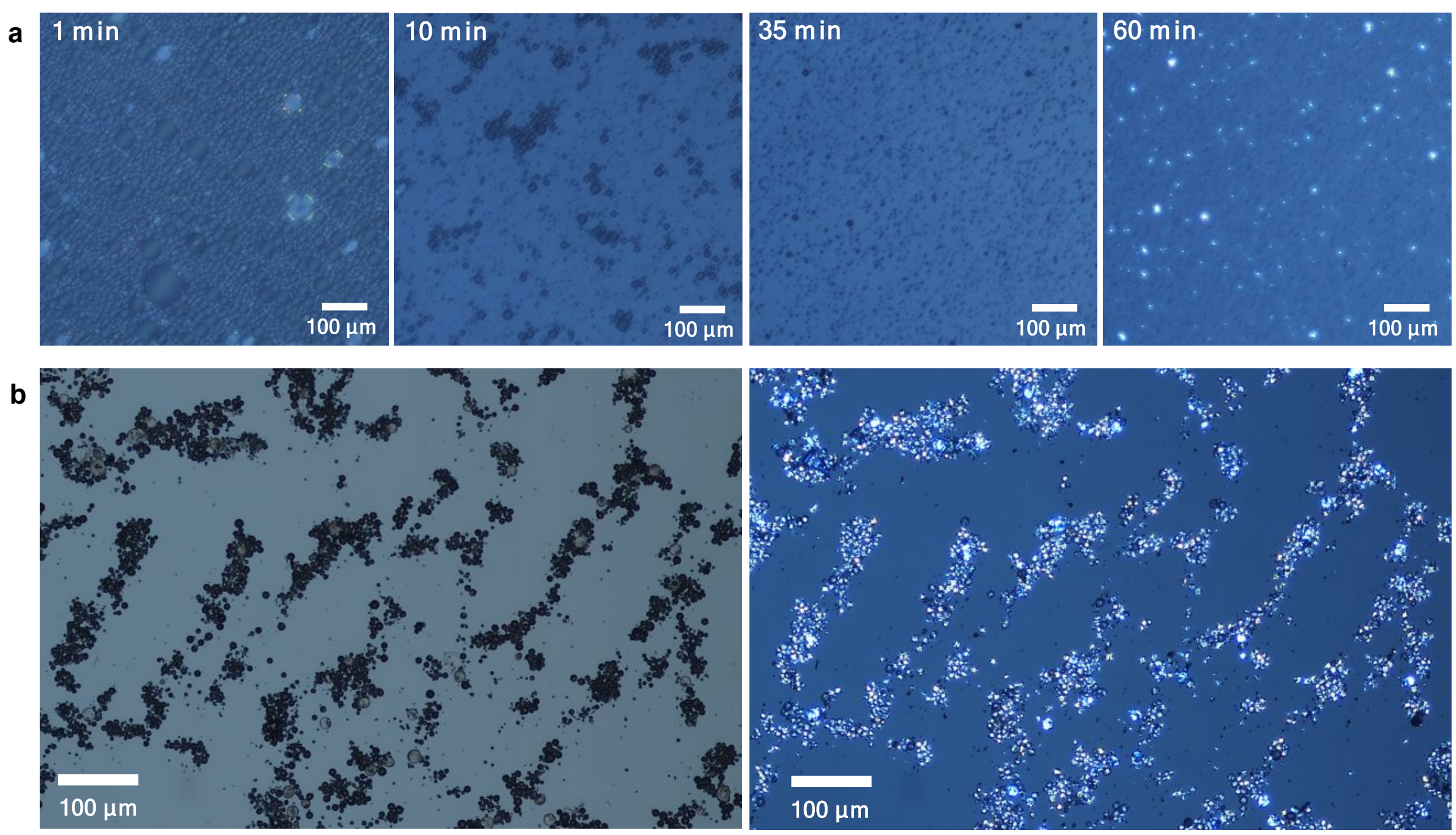

Figure S4: Time-lapse formation of Au/TiO_2 binary smectic supraparticles ($N_{Au}/N_{TiO2} \sim 0.05$). a) Cross-polarized optical microscopy images over time of the evaporation of emulsion droplets into supraparticles in water. b) Bright-field (left) and corresponding cross-polarized (right) optical microscopy images of the resulting dried supraparticles.

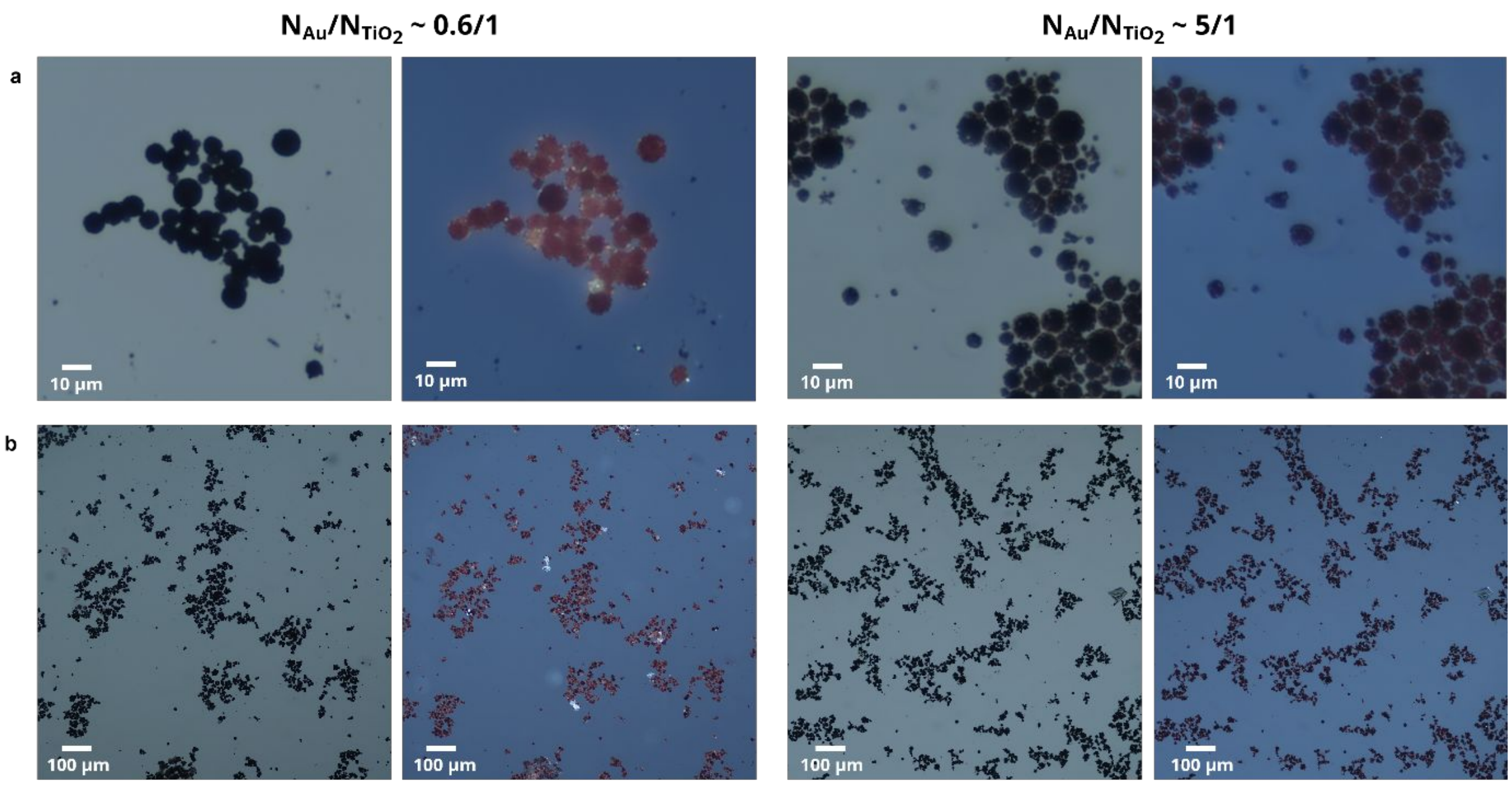

Figure S5: Characterization of Au/TiO_2 binary smectic supraparticles with high Au concentrations using polarized optical microscopy Bright-field (left) and corresponding cross-polarized (right) optical microscopy images of dried Au/TiO_2 supraparticles with particle ratio (N_{Au}/N_{TiO2}) of 0.6 and 5 at a) high and b) low magnifications.

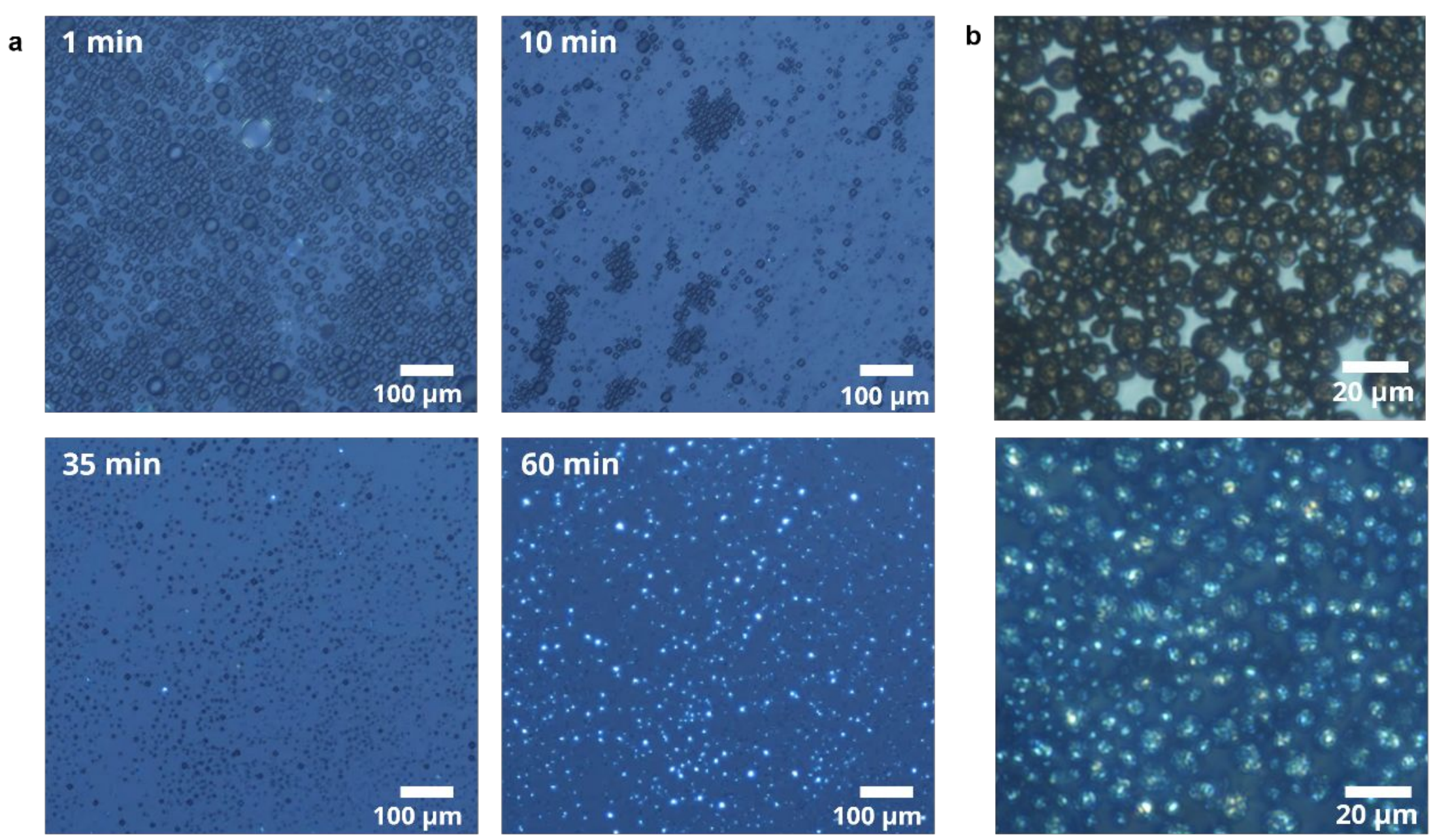

Figure S6: The formation of TiO_2 smectic supraparticles over time using polarized optical microscopy. a) Cross-polarized optical microscopy images of emulsion droplet evaporation into supraparticles over time. b) A bright-field (above) and corresponding cross-polarized (below) optical microscopy image of the TiO_2 supraparticles.

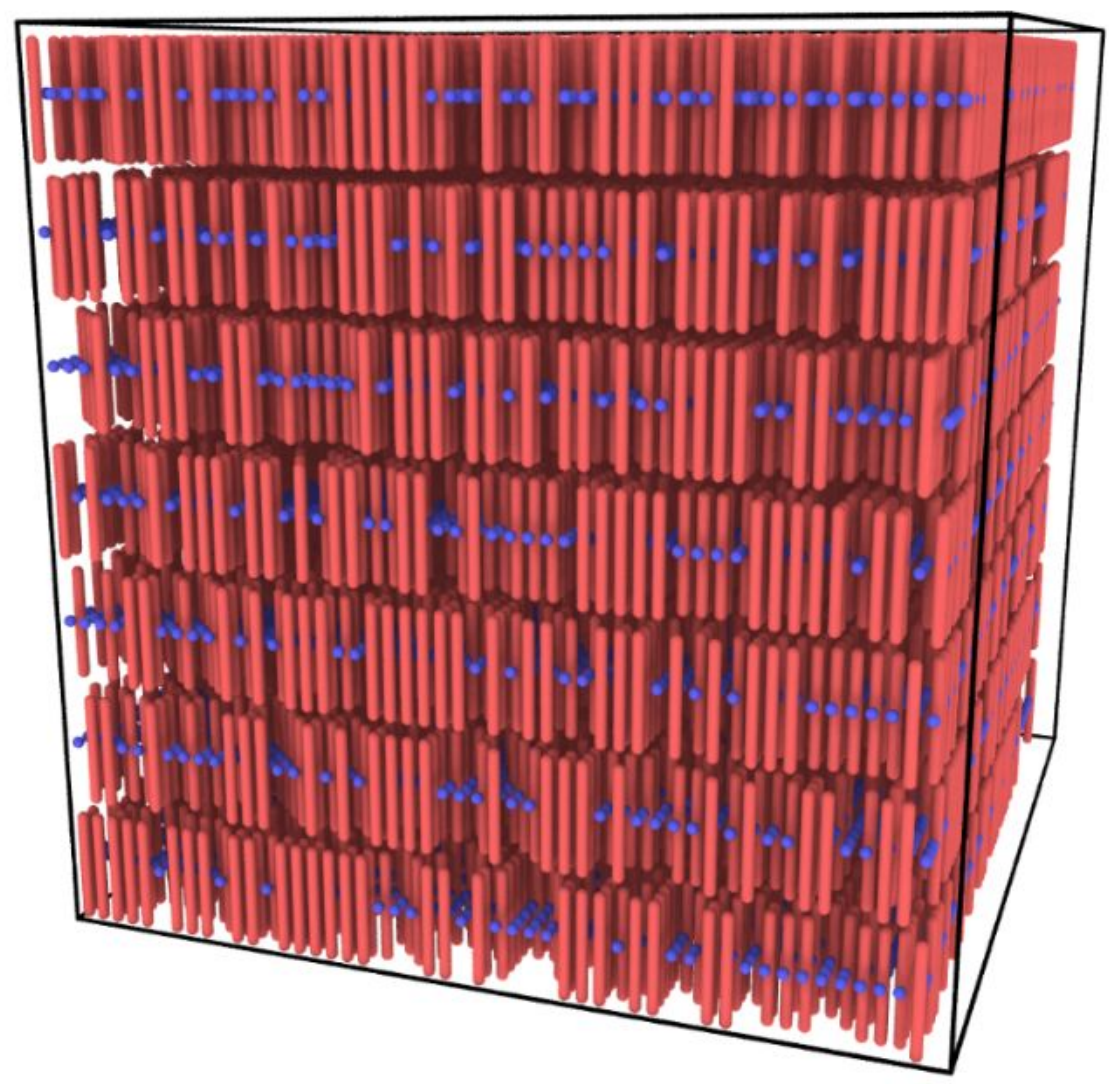

Figure S7: Typical configuration of the initial system configuration with equal numbers of rods (red) and spheres (blue). The rods are initially arranged on a $50 \times 50 \times 7$ grid, totalling 17500 particles. Rods are then randomly replaced by spheres to achieve the desired rod-to-sphere particle number ratio.

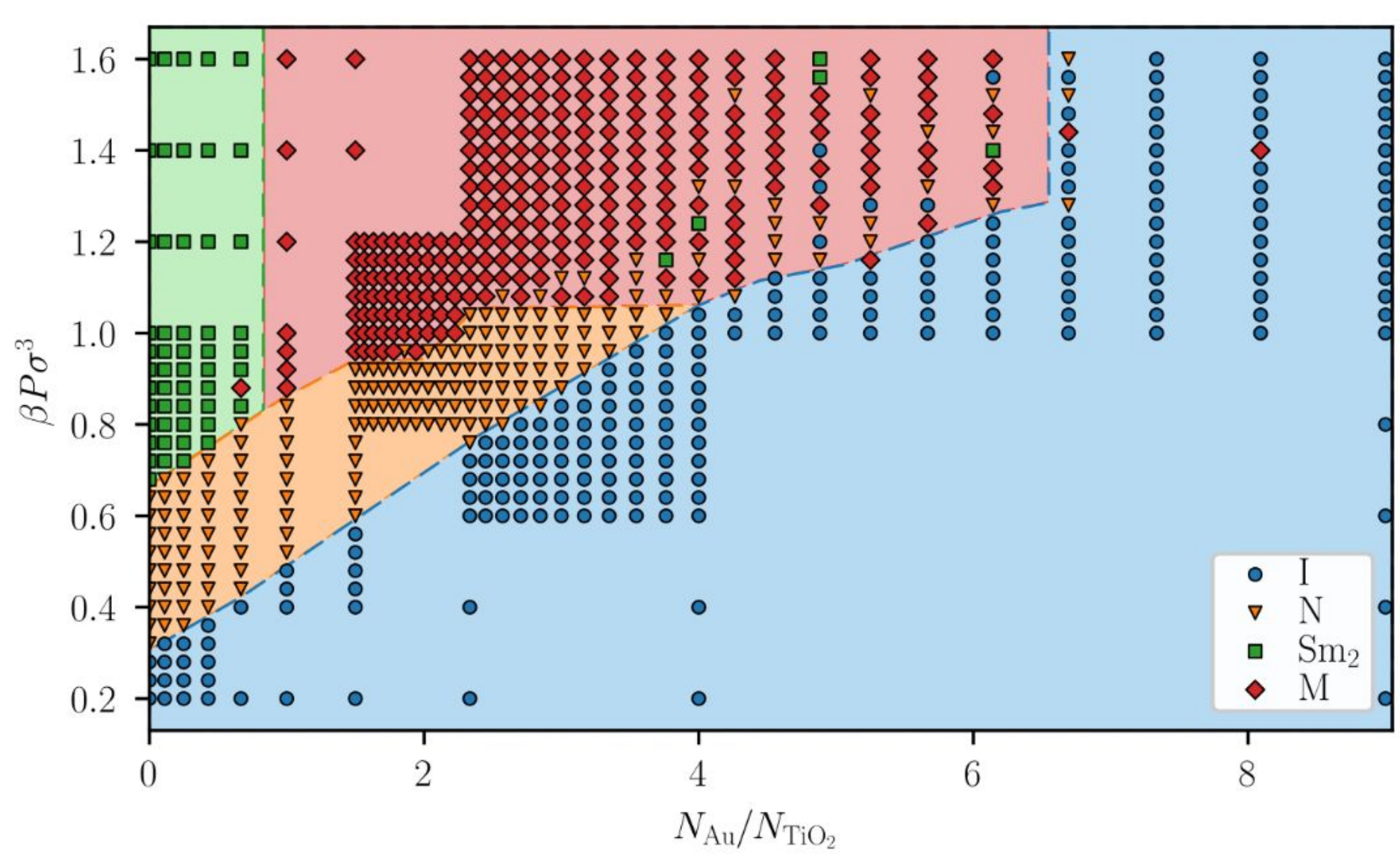

Figure S8: Phase diagram obtained from molecular dynamics simulations as a function of the particle number ratio N_{Au}/N_{TiO_2} and reduced pressure $\beta P \sigma^3$. The blue circles correspond to the isotropic phase (I), the orange triangles to the nematic phase (N), the green squares to the binary smectic phase (Sm_2), and red diamonds to the membrane phase (M).

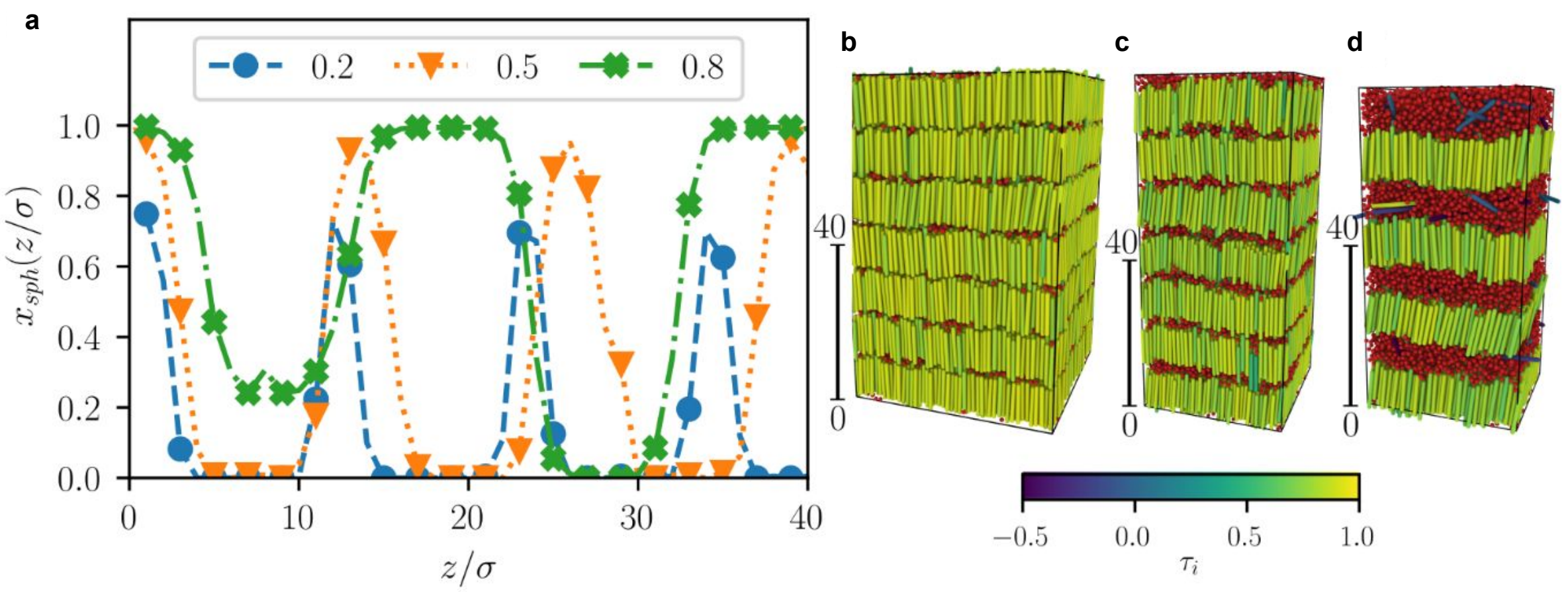

Figure S9: a) Local sphere composition $x_{\mathrm{sph}}(z) = N_{\mathrm{sph}}(z)/(N_{\mathrm{sph}}(z) + N_{\mathrm{rod}}(z))$ per slice of thickness σ as a function of z for different global sphere compositions (colours and markers) at fixed pressure $\beta P\sigma^3 = 0.16$. b-d) Representative snapshots for different sphere composition, b) $x_{\mathrm{sph}} = 0.2$, c) $x_{\mathrm{sph}} = 0.5$, and d) $x_{\mathrm{sph}} = 0.8$. Rods are coloured according to their local smectic order parameter τ_i, see color scale. At high sphere compositions, the system exhibit monolayers of rods and domains with an isotropic mixture of rods and spheres, as evidenced by the non-periodic local composition (green crosses in a).

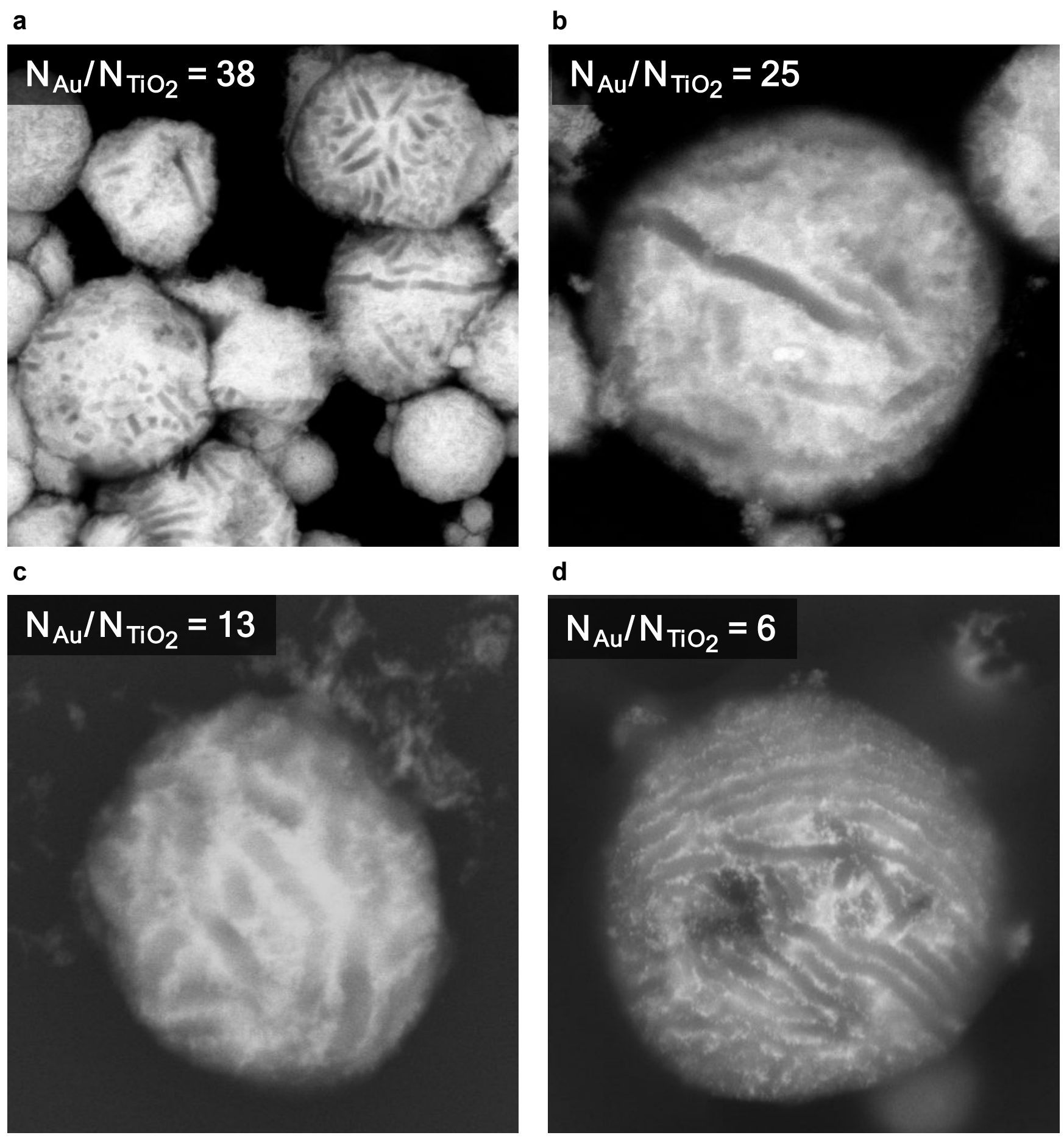

Figure S10: SEM images acquired using a BSE detector of Au/TiO_2 supraparticles with a N_{Au}/N_{TiO2} of a) 38, b) 25, c) 13 and d) 6, showing the presence of aligned TiO_2 rods (dark layers) surrounded by Au spheres (bright signal) even at high N_{Au}/N_{TiO2} .

N_{Au}/N_{TiO_2} = 2.5

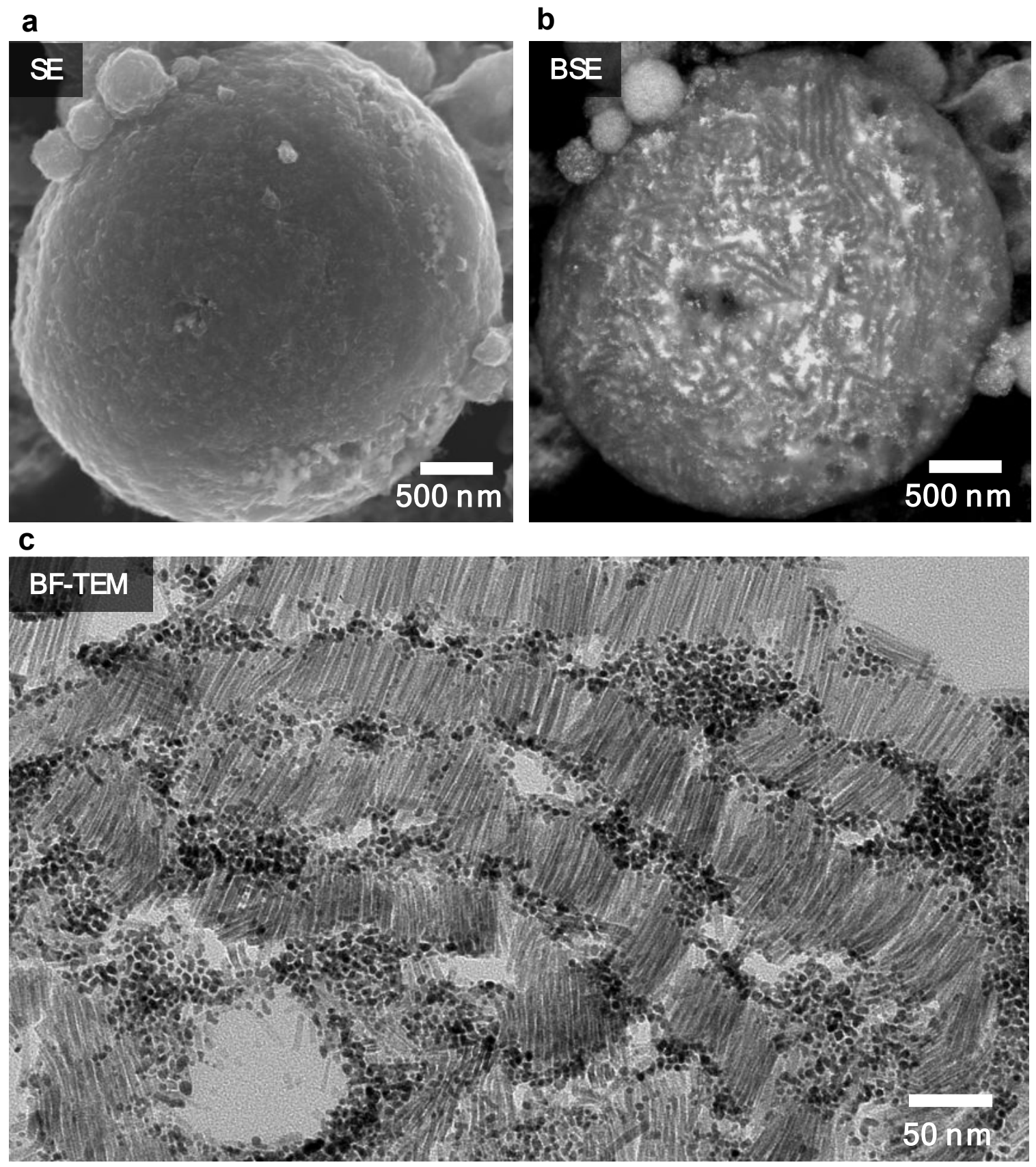

Figure S11: Structural characterization of Au/TiO_2 supraparticles with a N_{Au}/N_{TiO_2} of 2.5. a,b) SEM images of a representative Au/TiO_2 supraparticle captured with secondary electron (SE) and backscattered electron (BSE) detectors. c) BF-TEM image of an ultramicrotomy section of a Au/TiO_2 supraparticle.

3. References

(1) Gilbert, P. Iterative Methods for the Three-Dimensional Reconstruction of an Object from Projections. *J. Theor. Biol.* **1972**, *36* (1), 105–117. https://doi.org/10.1016/0022-5193(72)90180-4.

(2) *ImageJ User Guide - IJ 1.46r | Image Menu*. https://imagej.net/ij/docs/guide/146-28.html (accessed 2025-07-31).

(3) Thompson, A. P.; Aktulga, H. M.; Berger, R.; Bolintineanu, D. S.; Brown, W. M.; Crozier, P. S.; in ’t Veld, P. J.; Kohlmeyer, A.; Moore, S. G.; Nguyen, T. D.; Shan, R.; Stevens, M. J.; Tranchida, J.; Trott, C.; Plimpton, S. J. LAMMPS - a Flexible Simulation Tool for Particle-Based Materials Modeling at the Atomic, Meso, and Continuum Scales. *Comput. Phys. Commun.* **2022**, *271*, 108171. https://doi.org/10.1016/j.cpc.2021.108171.

(4) Bakker, H. E.; Dussi, S.; Droste, B. L.; Besseling, T. H.; Kennedy, C. L.; Wiegant, E. I.; Liu, B.; Imhof, A.; Dijkstra, M.; Blaaderen, A. van. Phase Diagram of Binary Colloidal

Rod-Sphere Mixtures from a 3D Real-Space Analysis of Sedimentation–Diffusion Equilibria. *Soft Matter* **2016**, *12* (45), 9238–9245. https://doi.org/10.1039/C6SM02162J.
